# Stress phenotyping of wild desert legume *Acacia senegal* with machine learning application and phytochemical characterization of bipinnate leaves

**DOI:** 10.3389/fnut.2026.1816860

**Published:** 2026-07-28

**Authors:** Karnika Singh, Rakhi Agrawal, P. M. Vighnesh, Ankur Lhila, Roshan Bagla, Pankaj Kumar Sharma, Mukul Joshi, Tanmaya Mahapatra

**Affiliations:** 1 Department of Biological Sciences, Birla Institute of Technology and Science, Pilani, Rajasthan, India; 2 Department of Computer Science and Information Systems, Birla Institute of Technology and Science, Pilani, Rajasthan, India; 3 Department of Electrical and Electronics Engineering, Birla Institute of Technology and Science, Pilani, Rajasthan, India

**Keywords:** *Acacia senegal*, automation, convolutional neural network (CNN), LC-MS/MS, machine learning, stress phenotyping, support vector machine (SVM)

## Abstract

Plants encounter multiple abiotic stresses. Among them, heat and drought stress play a substantial role in reducing the agricultural productivity of commercial plants. Hence, wild and underutilized plants can be a potential alternative as they are naturally tolerant to extreme climatic conditions and are a rich source of nutrition. Manual stress and disease detection is a laborious and expensive process, and hence automation in this field is required to reduce agricultural losses. This study evaluates the prediction and detection of abiotic stress in *Acacia senegal* bipinnate leaves, exploring various stress-induced changes using machine learning (ML) algorithms and biochemical analysis. *A. senegal*, an underutilized edible desert legume, was grown under controlled greenhouse conditions. After 2 months, these plants were segregated into groups and subjected to heat and drought treatments. Image acquisition was performed to obtain a dataset of 3,454 images of *A. senegal* leaves. Physiological parameters, such as fresh and dry leaf weight, shoot length, number of leaves, and biochemical assays like antioxidant assay (DPPH), total phenolic content (TPC), and total flavonoid content (TFC), were determined. LC-MS/MS analysis was conducted to identify over 50 phytochemical compounds. A hybrid model was developed consisting of a fine-tuned EfficientNet-based Convolutional Neural Network (CNN) followed by a Support Vector Machine (SVM) for the binary classification of *A. senegal* leaves. The model distinguishes between healthy and stress-affected unhealthy leaves and achieved an accuracy score of 86.6%. This report provides a significant lead toward stress phenotyping and prediction of a bipinnate leaf plant using ML algorithms. The overall study is useful to understand how the stress encountered by arid plants alters the nutritional quality.

## 1 Introduction

As the world population is growing, novel and innovative agricultural management strategies are required to ensure the sustainability of the agricultural sector in the future. According to reports, the world will have more than 9 billion people by 2050 (1). The task of feeding a huge population is challenging and will call for an integration of several concepts and methods from various academic fields. One of the major challenges is to ensure the highest feasible crop yields while reducing crop losses due to plant stresses, such as heat, drought, disease, and nutrient deficiencies. Crop yields of various conventional crops like rainfed rice, wheat, and maize are predicted to reduce by 47%, 40%, and 23%, respectively, by the year 2080 (2). Several aspects of food security, such as food availability and affordability, can be negatively affected if appropriate attention is not paid to controlling and preventing these losses (3).

Wild plants or crops growing in extreme environmental conditions of arid or semi-arid regions can have several uses and applications. These wild plants, such as *Acacia senegal* and *Prosopis cineraria*, are significant means of nourishment and revenue in numerous indigenous communities and hunger-prone areas. However, little focus has been given to wild food crops because of the lack of knowledge on their nutritional qualities or contribution to human diets, the poor dissemination of their food use, and the stigmatization complex caused by the label “famine food.” (4, 5). *A. senegal* is one of the important commercial and multifunctional agroforestry trees (6). It produces a very important industrial product called gum Arabic, which can be used for the manufacturing of various industrial products, such as food, beverages, cosmetics, and pharmaceutical products (7). The secondary metabolites extracted from *A. senegal* gum have anti-inflammatory, antimicrobial, antioxidant, anti-cancer, anti-diabetic, antiviral, and several other properties due to the presence of various bioactive compounds such as alkaloids, glycosides, flavonoids, polyphenols, arabic acid, phenolic acids, hydrocarbons, and fatty acids (8, 89). In tropical Africa, these trees maintain arid regions' ecosystems (9–11). In an agroforestry-based system, researchers have found that when these plants are cultivated together with various types of crops, the combined yields are higher than when these trees or crops are grown individually. These plants can also generate a significant amount of biomass with a minimum amount of water supply (12). Furthermore, the ethnoveterinary medicine potential of the *Acacia* genus is well-known in the African region, where a variety of plant parts, such as leaves, seeds, stem bark, roots, and pods, are utilized as pastes, infusions, and decoctions to treat wounds and bacterial and fungal diseases in livestock (13–15). The underutilisation of *A. senegal* is ascribed to a comparatively small number of seed sources, selection of better-performing varieties, lack of understanding of production processes, and stress phenotyping. Stress phenotyping is a crucial step to understand how these plants respond to harsh environmental conditions, and their morphological and physiological adaptations. Hence, for large-scale cultivation, there is a need to study these plants that are more adaptive and resilient to climate change and can help solve the problems related to food security as well as enhance agricultural productivity globally.

Precision agriculture (PA) combined with information technology can help farmers gather data and information to help them make decisions that will increase agricultural productivity. The application of these advanced technologies can lead to economic growth in the agricultural sector. There are several applications of PA, like plant disease detection, weed detection, pest detection, and crop yield prediction (16). As a significant amount of work, cost, and processing time are required for manual disease or stress detection, image processing becomes an essential tool to diagnose plant/crop stress or diseases from healthy and stress- or disease-affected unhealthy leaf images. Next-generation technologies like Machine Learning (ML) and Deep Learning (DL) can be used for early crop stress or disease prediction.

ML techniques offer a programmable and scalable method for data processing, particularly for the recently developed field of “plant stress analytics” (16). New investigations on high-throughput stress phenotyping with the use of image data collected from UAV-based platforms for the identification of weeds in plants like sunflower (*Helianthus annuus L*.) (17), maize (*Zea mays*) (18), and wheat (*Triticum aestivum L*.) (19) using ML algorithms have improved stress management techniques on a temporal and spatial basis. Moreover, DL models like Google Net, Alex Net, and Inception V3 were used for the detection of water stress in plants like okra (*Abelmoschus esculentus*), maize (*Zea mays*), and soybean (*Glycine max*) by using image processing and a dataset of 1,200 images (20). The ML approach has been used in previous studies to detect various stresses and diseases in plants that have broad leaf structure (21–24). The leaf structure of *A. senegal* is bipinnate, that is, it possesses leaflets, due to which the application of the ML algorithm for stress prediction in these plants becomes more demanding. This variation in leaf structure makes this study additionally challenging and unique.

*A. senegal* has nitrogen-fixing capabilities and contributes to restoring nitrogen-depleted soil through crop rotation (25). Legumes are well-acknowledged for their high content of bioactive compounds, such as phenolics, saponins, phytosterols, and carbohydrates, which contribute to the reduction of risks associated with oxidative agents, microbes, inflammatory diseases, diabetes, and cancer (26, 90). Plant secondary metabolites are multifunctional compounds that are generated by different biochemical pathways. These metabolites shield the plants from oxidative stress and infections. Phenols and flavonoids are the most crucial bioactive compounds that are responsible for the unique flavor, color, and bioactive potential of legumes. These compounds can be used in various industries, such as pharmaceuticals, food, and cosmetics. With one in five children under the age of five suffering from chronic malnutrition, legumes are increasingly seen as a potential superfood that might eliminate hunger and enhance health (27, 28). In this study, we performed DPPH (2,2-Diphenyl-1-Picrylhydrazyl), TPC, TFC, and liquid Chromatography-Mass Spectrometry (LC-MS) to see the effect of abiotic stress on the antioxidant activity and metabolite profile of *A. Senegal* and tried to correlate the biochemical and ML results to predict the effect of environmental stresses in these plants.

## 2 Methodology

### 2.1 Chemicals and reagents

DPPH was purchased from HiMedia (India). Methanol, hexane, DPPH, Folin Ciocalteu reagent, sodium carbonate, aluminum chloride, sodium nitrite, sodium hydroxide, and sulphuric acid were procured from Sigma-Aldrich Chemicals Company (United States), and quercetin dihydrate from SRL (India) and gallic acid were obtained from Yucca Enterprises (Mumbai, India).

### 2.2 Plant growth

*A. senegal* seeds were procured from Central Arid Zone Research Institute, Jodhpur, India, and the best seeds were selected manually for uniform germination. Acid treatment, often referred to as acid scarification, is a process that plays a significant role in seed germination, as it enhances the permeability of the seed coat to water and gases, breaks seed dormancy, and promotes seed germination. The seeds were treated with 15% H_2_SO_4_ for proper germination without any harm to the embryo. Uniformly germinated seeds were selected and sown to ensure the uniformity of the entire batch. The plants were grown under regulated greenhouse conditions at 28 °C with a photoperiod of 16 h light: 8 h dark for 60 days.

### 2.3 Stress treatment and physiological analysis

*A. senegal* is a desert plant that has drought-tolerant properties. Drought stress treatment was started on day 45 for the best physiological changes and results. Heat stress and heat+drought (H+D) stress treatments were started on day 61, and all these stresses were stopped on day 85 of plant growth. The heat treatment of 45–50 °C was given to plants for a total of 4 h daily. Physiological parameters, such as fresh and dry leaf weight, number of leaves, shoot length, and number of branches were calculated.

### 2.4 Plant leaf collection

Leaves were collected from the stress-treated plants at different time intervals, that is, 5th, 10th, 15th, 20th, and 25th days. These leaves were separated from the leaf stalks, and the fresh weight of these leaves was measured. Afterwards, these leaves were wrapped in foil and stored overnight at 4 °C. The next day, these leaves were dried using the convection feature in the microwave at 40 °C for 4–5 h. Dry leaf weight was calculated after drying the leaves completely. Then these dried leaves were crushed into a fine powder using a mortar and pestle, followed by the measurement of crushed leaf weight. These crushed leaf samples were labeled and stored carefully at 4 °C for further biochemical extractions and analysis.

### 2.5 Imaging and dataset acquisition

Image acquisition included capturing images with appropriate angles, proper lighting, and depth to obtain images of higher quality and resolution. The imaging of control plants grown at 28 °C and other optimum conditions without any abiotic stress was started on day 31 and was performed once a week up to the completion of day 60. The imaging of stress-treated plants was done every 5th day after day 60 of plant growth and up to day 85. The leaves at the fourth position from the bottom to the top were considered throughout the imaging period to ensure uniformity of the dataset. For imaging, a Canon EOS R6 Mk 2 camera with a 24.2 MP resolution, 24–105 mm f4 lens, and 105 mm focal length was used. All photos were taken in auto mode with autofocus and stabilization in outdoor light and cropped in a 6:6 ratio within the camera. These are top-down images with black cloth as a background.

### 2.6 Dataset pre-processing

The dataset (91) consists of 3,454 images, of which 2,105 images are healthy while 1,349 images are stress-affected unhealthy. The labeling was done without any bias from the results of other leaves in the parent plant and based on the presence of spots, discolouration, and damage on the leaves. Various pre-processing techniques have been applied to the images, as discussed below.

#### 2.6.1 Cropping

**Figure 1:**
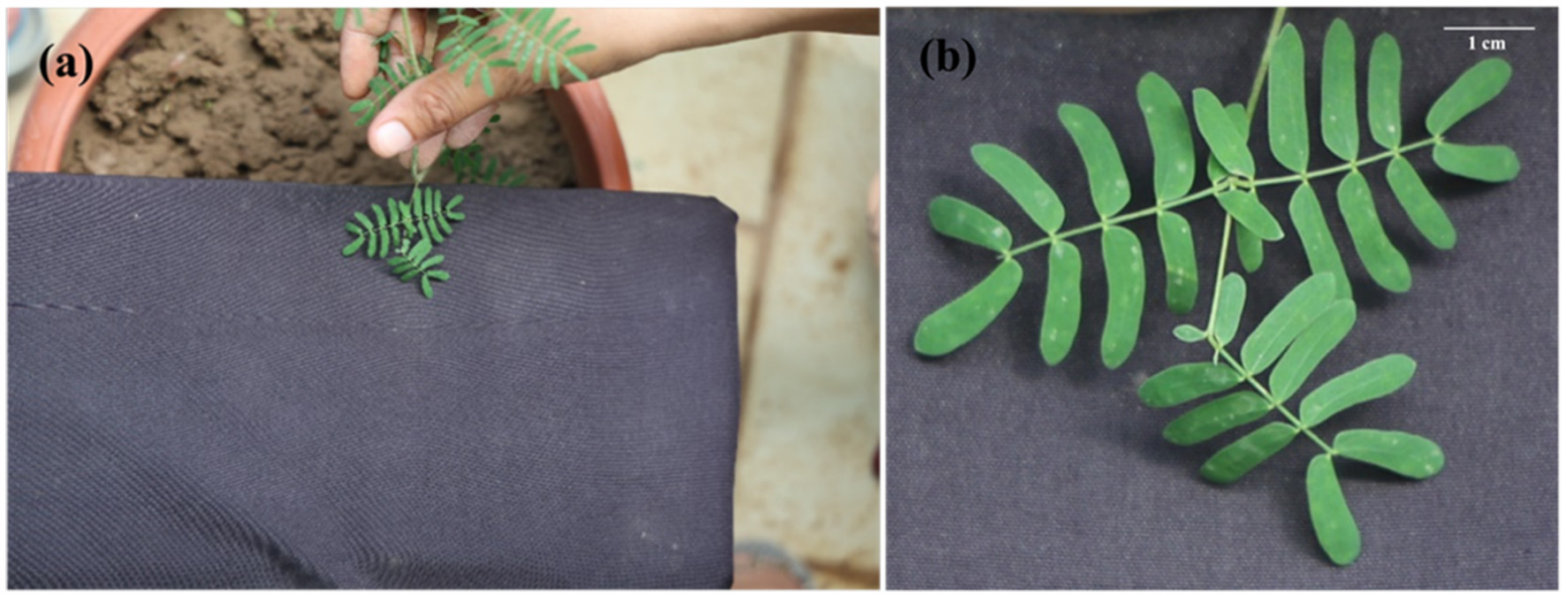
Image cropping, **(a)** sample image before cropping, **(b)** sample image after manual cropping.

The images have been manually cropped to focus on the plant leaves and remove unnecessary background. Freestyle cropping was done, and later the images were resized to a fixed size. Figure 1 illustrates the results of cropping.

#### 2.6.2 Color transformation, YG filtering, and background removal

**Figure 2:**
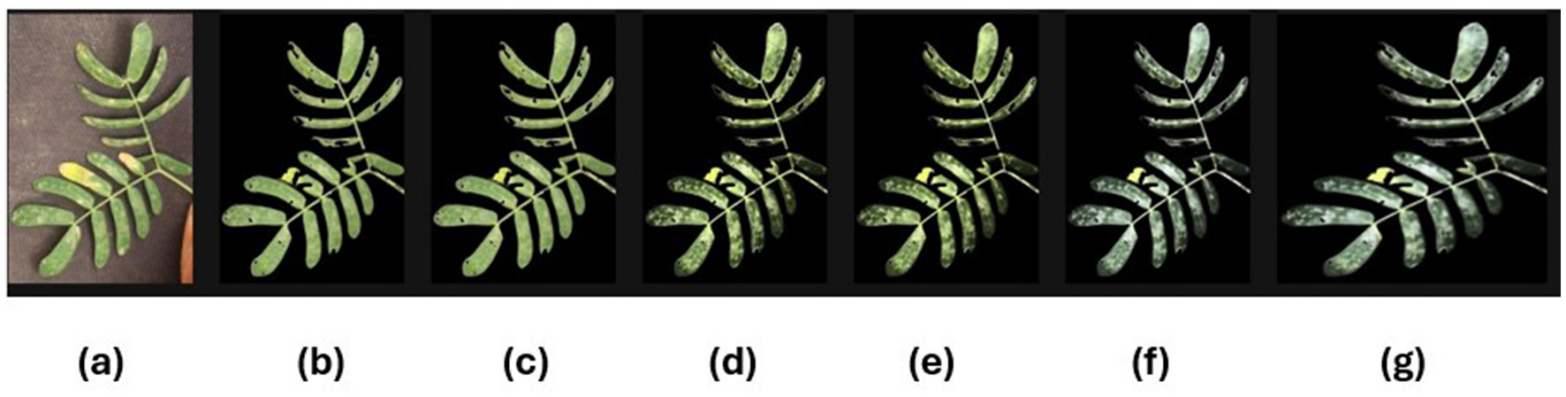
Workflow of preprocessing images **(a)** original manually cropped image of the leaf **(b)** HSV conversion, leaf image with no background and yellow-green filtering **(c)** cropped image to foreground **(d)** histogram equalization was done to enhance image contrast **(e)** bilateral filter is applied to reduce the noise **(f)** image normalization to rescale the raw 0–255 pixel values **(g)** resized image of the leaf (224 × 224 pixels).

Images were acquired in an RGB color space where three channels are used to depict the proportions of red, green and blue. To allow us to isolate the leaf images from both the background and non-leaf artifacts (such as fingers holding the stem or debris), we have converted the RGB color space to HSV color space where the three channels represent Hue, Saturation, and Value, respectively. The hue represents colors expressed as angles corresponding to their region in the color wheel. Saturation tells us the quantity of the color, while value refers to the brightness or the amount of white light present. By defining precise threshold boundaries within the Hue channel, a Yellow-Green (YG) filtering technique was applied. While the saturation and value channels allowed us to select the pixels corresponding to the leaves and discard the background, the YG filter specifically isolated the distinct hue range of leaf tissue. Unhealthy leaves showed yellow spotting after stress treatment, and healthy leaves were green without any spots. To isolate these features from the image, we created a mask on the image, taking the hue values in the range of 60–200° with saturation and brightness varying from 50 to 255. The above step removed the background and its texture, which may come across as noise when training ML models (Figure 2).

#### 2.6.3 Crop to foreground

While the initial manual cropping narrowed the focus to the leaf area, unwanted elements like fingers and stem debris remained. Following the Yellow-Green (YG) filtering stage, these non-leaf artifacts were successfully isolated and removed, leaving only the pure leaf tissue against a clean background. This precise color segmentation enabled an automated, tight foreground crop around the remaining leaf pixels, eliminating redundant background space and minimizing the input noise before the final normalization and resizing stages.

#### 2.6.4 Histogram equalization

Histogram equalization is a method that uses the image histogram and accordingly adjusts each pixel value to give a more uniform distribution. This helps to increase the image contrast. We applied this method to allow the images to have more visible features for the model to detect, allowing for better accuracy. The formula is as follows:

$$\begin{array}{l} s_{k}=\left(L-1\right)\overset{k}{\underset{j=0}{\sum }}p\left(r_{j}\right) \end{array}$$

#### 2.6.5 Bilateral filtering

Bilateral filtering (29) is a non-linear image processing technique that helps preserve edges while smoothing images and reducing noise. We applied this filtering method to minimize noise while maintaining the integrity of edges. This approach was particularly useful for protecting the spots and edges while smoothing the leaves. The pixel values for bilateral filtering are calculated as follows:


$$\begin{array}{l} I_{filtered}\left(x\right)=\frac{1}{W_{p}}\underset{x_{i}\in \Omega }{\sum }I\left(x_{i}\right)\cdot f_{r}\left(I\left(x_{i}\right)-I\left(x\right)\right)\cdot f_{s}\left(\left|x_{i}-x\right|\right) \end{array}$$

Here, I(x) is the intensity of the pixel at x, Ω is the neighborhood of pixel x, f_r_ is the range kernel function, f_s_ is the spatial kernel function, and W_p_ is the normalization factor defined as:


$$\begin{array}{l} W_{p}=\underset{x_{i}\in \Omega }{\sum }f_{r}\left(I\left(x_{i}\right)-I\left(x\right)\right)\cdot f_{s}\left(\left|x_{i}-x\right|\right) \end{array}$$

#### 2.6.6 Image normalization and image resizing

Prior to spatial adjustments, image normalization was performed to rescale the raw 0–255 pixel values into a stable, bounded floating-point range. Normalizing at this stage prevents mathematical distortion during interpolation and ensures the loss landscape remains uniform, accelerating gradient descent while preventing vanishing or exploding gradients. Following normalization, the images were resized to a standardized resolution of 224 × 224 pixels. This specific dimension matches the native input specifications required by industry-standard backbone architectures like ResNet, DenseNet, and EfficientNet. Replicating this exact sequence aligns the dataset with pre-trained ImageNet distributions, enabling the transfer learning models to accurately extract leaf features right from the start of training.

#### 2.6.7 Data augmentation

After applying various image pre-processing techniques, the image underwent a random combination of multiple transformations. These changes included horizontal and vertical flipping, random rotations between −30 and 30 degrees, brightness adjustments, zooming, and adding Gaussian noise. For example, one copy might be flipped, rotated, and given noise, while another copy might only change in brightness and zoom. This mixed approach creates realistic variations and stops the model from overfitting, helping it learn better. This process resulted in an expanded dataset size of 16,000 images.

### 2.7 Model

**Figure 3:**
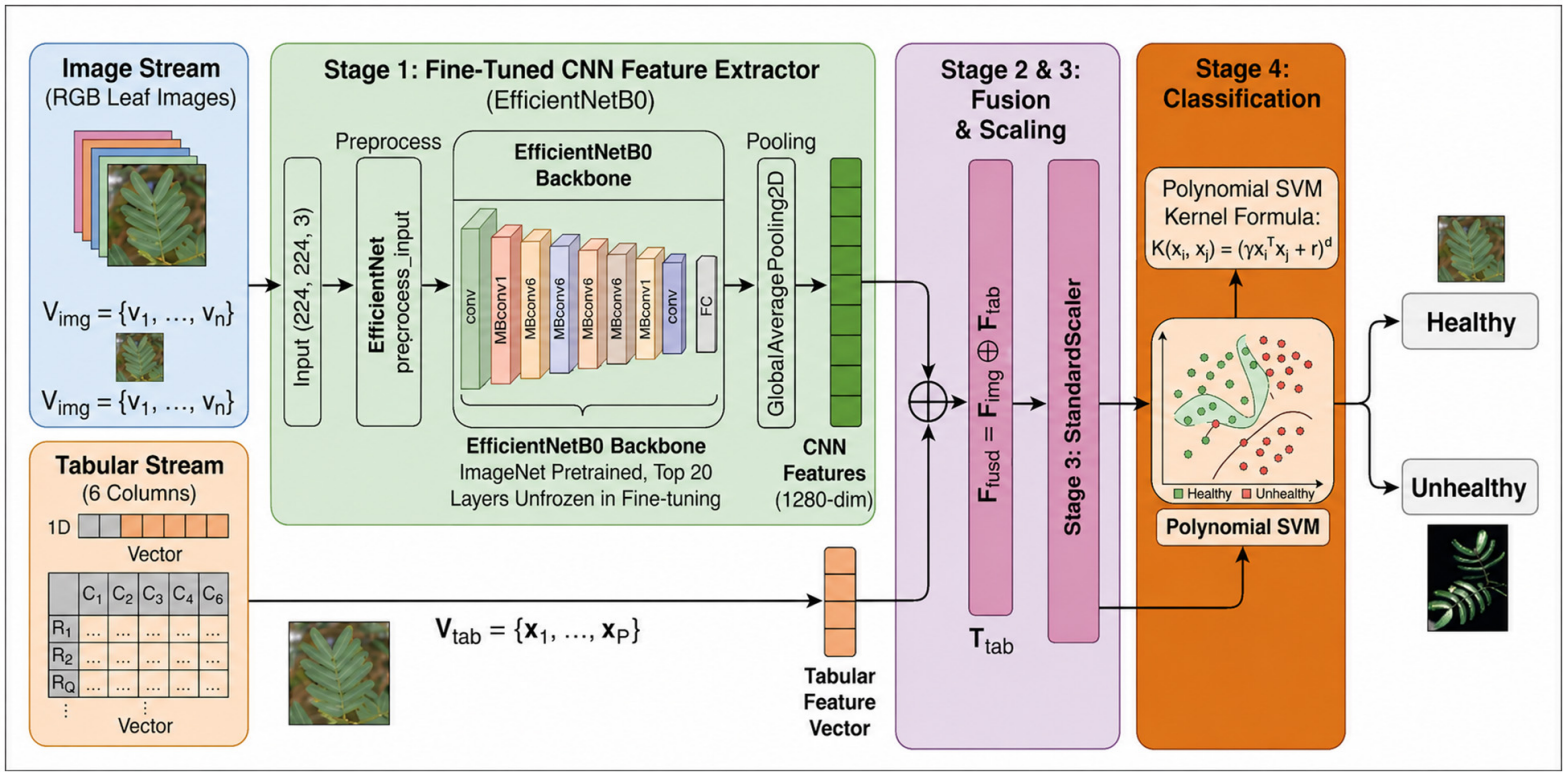
Architecture of hybrid custom CNN-SVM model. This figure was generated by Google Gemini 1.5 Flash model and the input prompt can be found in Supplementary File 1.

We employed a hybrid custom deep learning and machine learning framework consisting of an *EfficientNetB0-based Convolutional Neural Network (CNN)* as a feature extractor followed by a *Support Vector Machine (SVM)* classifier for the binary classification of *Acacia senegal* leaves into healthy and unhealthy categories. The hybrid approach combines the powerful feature-learning capability of deep convolutional networks with the strong generalization performance of SVMs in high-dimensional feature spaces. Figure 3 illustrates the architecture of our hybrid custom CNN-SVM model. The implementation details of our model are publicly available via a GitHub repository.^1^

The CNN component is based on the EfficientNetB0 architecture, which scales network depth, width, and input resolution using a compound scaling strategy to achieve high classification performance with relatively few parameters. The model was initialized with weights pre-trained on the ImageNet dataset and subsequently adapted to the *Acacia senegal* leaf classification task through transfer learning. The input images were resized to 224 × 224 pixels and represented in three channels as required by the EfficientNetB0 architecture.

To adapt the pre-trained model to the target dataset, the original classification head of EfficientNetB0 was removed, and a single-neuron sigmoid output layer was introduced immediately after the Global Average Pooling (GAP) layer, which was used to convert the final feature maps into a compact feature vector (1280-dim). During fine-tuning, the top 20 layers of EfficientNetB0 were unfrozen, while the remaining layers retained their pre-trained weights. This enabled the network to learn domain-specific visual characteristics of *Acacia senegal* leaves while preserving the general image representations learned from ImageNet. A classification head consisting of a dropout layer with a rate of 0.50 was initially employed during fine-tuning. However, for our hybrid custom CNN–SVM model, the features extracted from the GAP layer were used directly, bypassing the classification head.

EfficientNetB0 incorporates batch normalization layers throughout the network, which improve training stability and accelerate convergence by reducing internal covariate shift. To further improve generalization and reduce overfitting, extensive data augmentation was applied during training, including random geometric and intensity transformations. The Adam optimizer was used during fine-tuning due to its adaptive learning rate capabilities. Hyperparameter tuning was performed to identify the optimal learning rate and training configuration for the network.

After fine-tuning, the CNN was used solely as a feature extractor. The feature vectors generated by the GAP layer were *concatenated with the tabular data* and then were supplied to an SVM classifier, which performs the final binary classification. The tabular data in our study captures environmental conditions, such as *heat* and *drought*, to which *Acacia senegal* leaves were subjected to on a weekly basis. SVMs are particularly effective in high-dimensional feature spaces and often exhibit better generalization than fully connected neural network classifiers when training data are limited. The SVM identifies an optimal separating hyperplane that maximizes the margin between healthy and unhealthy leaf classes.

The SVM classifier employs L2 regularization implicitly through the cost parameter (C), which controls the trade-off between margin maximization and classification error. To address class imbalance, balanced class weighting was incorporated during training. Hyperparameter optimisation identified a Polynomial kernel as the most effective choice, indicating that the deep features extracted by EfficientNetB0 were sufficiently discriminative to achieve linear separability when mapped to a higher-dimensional space. The optimal value of the regularization parameter was found to be C = 1.0, while the gamma parameter was set to auto. In this hybrid framework, regularization is achieved through a combination of dropout and data augmentation during CNN fine-tuning, followed by SVM margin regularization through the C parameter during classification.

#### 2.7.1 Model evaluation

The performance of our hybrid custom CNN-SVM model was evaluated using *Stratified K-Fold Cross-Validation*. Stratification ensures that each fold preserves the original class distribution of the dataset, thereby reducing sampling bias. The dataset was divided into fivefolds, with fourfolds used for training and onefold used for validation in each iteration. This process was repeated five times so that every sample served as part of the validation set exactly once. The final comprehensive performance metrics, including *AUC-ROC, Accuracy, Precision, Recall* and *F1-score*, were obtained by averaging the results across all folds, providing a robust estimate of the model's generalization capability. Additionally, we have computed the 95% confidence interval, along with its statistical significance.

#### 2.7.2 Hyperparameter optimisation

Hyperparameter tuning was conducted using a Grid Search strategy separately for the feature extractor and binary classifier. The search process for the custom CNN evaluated different combinations of CNN fine-tuning, while the same, combined with fivefold stratified cross-validation, was used to find the optimal SVM parameters to identify the configuration yielding the highest validation performance.

The principal hyperparameters explored were (i) *learning rate:* values of 0.000005, 0.00001, 0.0001, and 0.001 were investigated to balance convergence speed and training stability during EfficientNetB0 fine-tuning, (ii) *dropout rate:* a dropout rate of 0.50 was employed in the temporary classification head during fine-tuning to improve generalization and reduce overfitting, (iii) *number of trainable layers:* different fine-tuning configurations were examined, with the optimal model obtained by unfreezing the top 20 layers of EfficientNetB0, (iv) *SVM regularization parameter (C):* values of 0.01, 0.1, 1, and 10 were evaluated to determine the optimal balance between margin maximization and classification accuracy, (v) *kernel function:* linear, polynomial, sigmoid and radial basis function (RBF) kernels were compared, (vi) *gamma parameter:* the values scale and auto were evaluated during optimization to determine the most suitable influence radius of training samples.

Our hybrid custom CNN-SVM model combines the representational power of a state-of-the-art CNN with the robust decision boundaries of an SVM classifier, resulting in a computationally efficient and accurate framework for *Acacia senegal* leaf health classification.

### 2.8 Preparation of leaf extract

The fine powder of crushed leaf samples was defatted with hexane in a 1:5 (w/v) ratio for 1 h in an orbital shaker incubator at room temperature. This was followed by centrifugation at 3,000 *g* for 10 min. The supernatant was decanted, and the pellet was extracted two more times. The hydrophobic compounds were separated in the hexane extract, and the pellet was dried at room temperature. This dried pellet was extracted twice in a 1:5 (w/v) ratio using 80% methanol by the same process mentioned above. The supernatant was filtered using Whatman filter paper (No. 1). The supernatants were pooled and stored at 4 °C for further biochemical analysis.

### 2.9 Total phenolic content (TPC)

The TPC was calculated by the Folin–Ciocalteu method described (30–32) with a few modifications. In Brief, 100 μl of the leaf extract was mixed with 400 μl of 7.5% sodium carbonate and 500 μl of the Folin-Ciocalteu Reagent (FCR). The mixture was shaken gently and incubated at room temperature for 30 min, and the absorbance was calculated at 765 nm. Gallic acid was used as standard. A calibration curve was plotted using standard gallic acid. The gallic acid standard curve was made with different concentrations (50–250 μg/ml), and the TPC values were expressed as micrograms of gallic acid equivalents (GAE) per gram of sample. All tests were performed in triplicate.

### 2.10 Total flavonoid content (TFC)

The TFC was determined by the aluminum chloride colorimetric assay described by John et al. (33). Briefly, 250 μl leaf extracts were taken in a test tube. To this test tube, 75 μl 5% NaNO_2_ was added, followed by 75 μl 10% AlCl_3_ after 5 min. This was followed by the addition of 500 μl 1 M NaOH, and the volume was made up to 2.5 ml with distilled water. The solution was shaken at each step to mix the reagents well, and absorbance was taken at 510 nm. Quercetin was used as standard. A calibration curve was plotted using standard quercetin. The standard curve of quercetin was prepared by taking various concentrations (200–1,000 μg/ml), and TFC values were expressed as milligram of quercetin equivalents per gram of sample. All tests were performed in triplicate.

### 2.11 LC-MS/MS analysis of plant leaf extracts

The crude methanolic extracts of four plant leaf samples were concentrated with the help of a desiccator and sent to the Sophisticated Analytical Instrument facility at CSIR-Central Drug Research Institute, Lucknow, for LC-MS analysis. This process was performed in accordance with Venuprasad et al. (34) and Chaudhary et al. (35). The samples were analyzed by LC–MS; HPLC-TQD Mass Spectrometer (Waters Corporation, Milford, CT, USA). Samples were separated on an Accucore C-18 column (150 mm × 4.6 mm, 6 μm, Thermo Scientific, USA). The mobile phase consisted of Acetonitrile (ACN) (A) and 5 mM ammonium acetate (B). The flow rate was 1.0 ml/min, injection volume 10 μl, and column temperature 35 °C. A photodiode-array (PDA) detector was used to record the spectral data from 200 to 600 nm. Mass spectrometry data using electrospray ionization were collected in both positive and negative ionization modes, covering a mass range of *m/z* 100–750. The detection parameters were as follows: gas flow 50 L/h; ion spray voltage 3.5 kV; collision energy 20 V; and desolvation temperature 350 °C. The mass spectrum fragments were evaluated by using mzCloud-Advance mass spectral database as well as the reported relevant literature (Supplementary Tables S2–S5).

### 2.12 DPPH: radical scavenging assay

The antioxidant activity of plant leaf extracts was tested by the DPPH assay. This assay was carried out in accordance with (36). The DPPH methanolic solution is purple/violet in color, which fades to pale yellow in the presence of antioxidants, and the loss in absorbance is measured at 517 nm. A 100 μM DPPH solution was prepared in methanol, and 290 μl of this solution was mixed with 10 μl of individual leaf extract. The reaction was carried out in a 96-well microplate, incubated in the dark at room temperature for 1 h, and the absorbance was measured at 517 nm using a microplate reader (Thermo Scientific Multiskan GO). All the tests were performed in triplicate. The percentage DPPH radical scavenging activity was calculated by the following equation:


$$\begin{array}{l} Inhibition\%=\frac{A_{c}-A_{s}}{A_{c}} \end{array}$$

where A_s_ is the absorbance of the sample and A_c_ is the absorbance of the control. Solution without the sample (leaf extract or phytochemical) was taken as the control. Ascorbic acid was used as a standard. A calibration curve was plotted using standard ascorbic acid. The standard curve of ascorbic acid was prepared by taking various concentrations (50–200 μg/ml). The results were expressed as EC_50_ (μM) obtained by plotting a curve between concentration and inhibition percentage. EC_50_ is the effective concentration necessary to get 50% inhibition. The lower the EC_50_ value, higher will be the antioxidant activity.

### 2.13 Statistical analysis

The data were presented as mean ± SD. Statistical significance was assessed using two-way analysis of variance (ANOVA) followed by Tukey's *post hoc* test for multiple comparisons, using GraphPad Prism software (Version 10.3.1, GraphPad Software, USA). A *p*-value < 0.05 was considered statistically significant (*p* > 0.05 ns; *p* ≤ 0.05 *; *p* ≤ 0.001 ^***^; *p* ≤ 0.0001 ^****^). All the experiments were technically conducted in triplicate to ensure adequate scientific accuracy of the experiments.

## 3 Results and discussion

### 3.1 Plant growth and physiological studies under stress conditions

**Figure 4:**
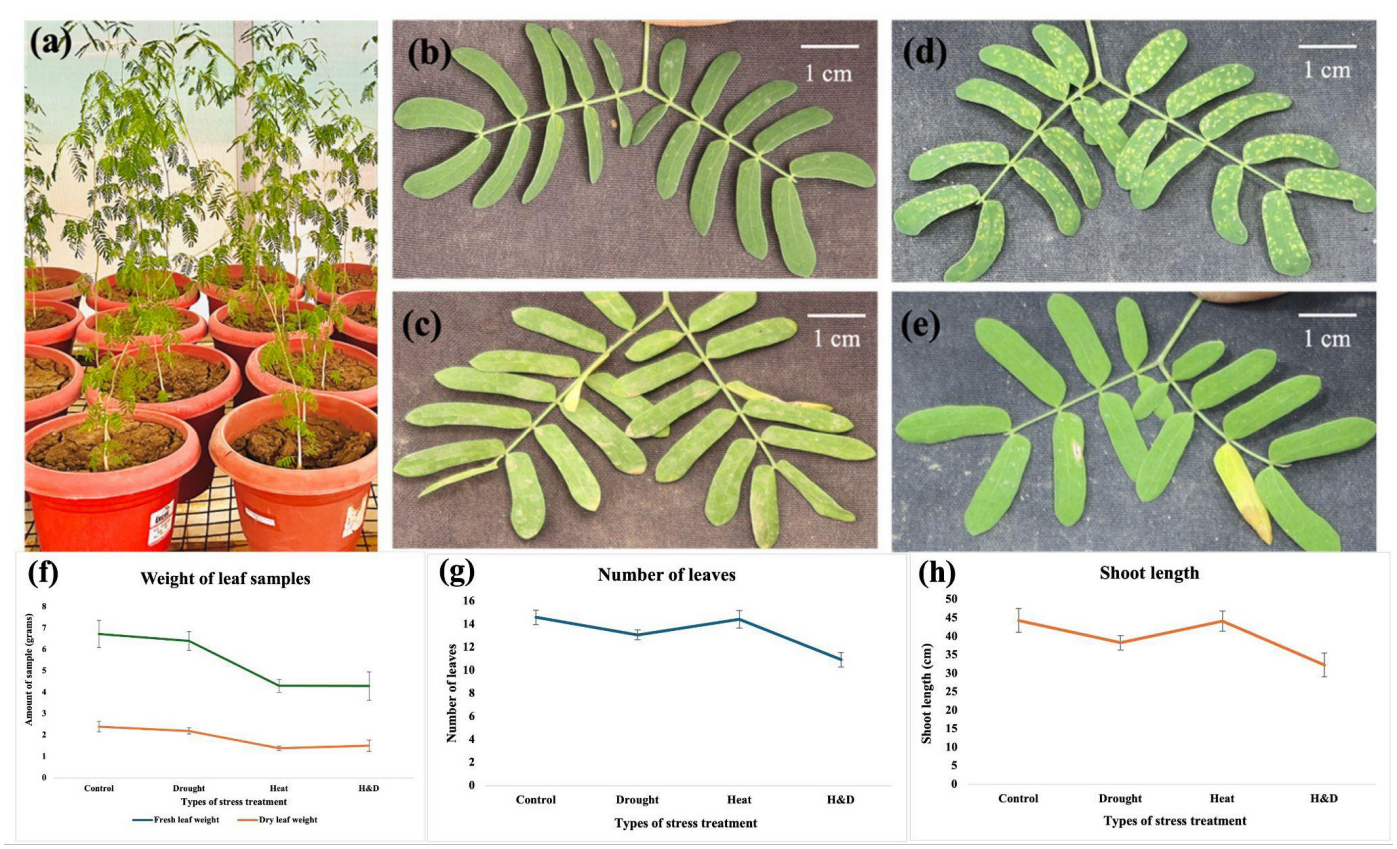
**(a)** *Acacia senegal* plants growing under controlled greenhouse conditions, **(b)** control plant leaf, **(c)** drought treated plant leaf, **(d)** heat treated plant leaf, **(e)** H+D treated plant leaf (25th day of stress treatment), **(f)** amount of fresh and dry leaf weight **(g)** number of leaves and **(h)** shoot length of control, drought, heat and H+D plants. Bars shown in the graph represent standard deviation.

The effect of stress treatments in control and stress-treated plants is visible in Figure 4. The majority of control plants were healthy and had no discolouration or damage on their leaves. However, for fifth day of stress treatment, the plants belonging to the drought, heat, and H+D category had 22, 10, and 22 discolored; 9, 7, and 9 damaged; zero spotted; and 4, 18, and 4 healthy leaves, respectively, out of 35 plants for each stress category. These same plants showed an increase in the number of unhealthy leaves on the 25th day of stress treatment. Interestingly, 24 out of 35 plants in the heat-treated category had significant yellow spotting on their leaves on the 25th day of stress treatment, but no spotting was observed when the plants were subjected to drought or H+D stress. Moreover, branching was not observed in the control, drought, or H+D plants, and was only seen in two heat-treated plants on the 25th day of stress treatment in the entire batch. Furthermore, it can be seen that the amount of fresh and dry leaf weight decreases in the stress-treated plants as compared to control plants. The shoot length and number of leaves per plant were observed to be decreased in stress-treated plants as compared to control plants, specifically in drought and H+D-treated plants (Figure 4).

The difference between the leaf weight of control and drought-treated plants was not very significant. Plants may adapt to sustain their growth and frequently become more drought resistant in response to water constraints through morphological adaptations, physiological regulations, and changes in the allocation of biomass. In order to survive in arid conditions, drought-tolerant plants frequently devote more resources to root growth than shoot growth (37). The root-to-shoot ratio is essential and can help plants to adapt in water-stress environments. However, the root adaptation to drought is variable across different species (38, 39), and the drought stress response of *A. senegal* roots has not yet been studied. Less restricted root growth, a rapid rate of root growth compared to shoot growth, or a decrease in shoot growth without affecting root growth can lead to changes in allocation of biomass of the plants (39–42). According to studies, mechanisms specific to species or genotypes regulate biomass partitioning to organs, affecting the capability of plants to adapt to changing environmental conditions (37, 43–45). The outermost surface of the plant leaves is protected from environmental stresses like thermal stress, water stress, and mechanical damage, as well as harmful radiation, by a hydrophobic extracellular layer known as the cuticle membrane (CM) (46, 47). Moreover, studies showed that there is no substantial variation in cuticular wax deposition in young, growing, and mature leaves of arid plants, which indicates that the capability of wax deposition in mature plants is not reduced or lost. Higher wax deposition restricts water permeability and water loss, leading to drought tolerance (48). In *A. senegal*, major xeromorphic modifications to water stress are densely packed long palisade mesophyll cells and a thick phyllode. Scleromorphic adaptations include thick cuticle, abundant lignified parenchyma and sclerenchyma cells, and adaptation to low phosphorus availability (49). These unique modifications support the plant's survival in drought stress (Figure 4).

There was a significant decrease in the leaf weight of heat and H+D plants as compared to control and drought-treated plants. The magnitude of protection provided by CM is dependent on the quantity and configuration of the cuticle membrane's constituents such as polysaccharides, wax, phenolics, and polyester cutin matrix (47–50). It has been observed that waxes are crucial for preventing water loss, whereas phenolic compounds are extremely effective in regulating the mechanical properties of cuticle membranes (51, 52). In general, wax production is influenced by temperature in several plants; higher amounts of wax are produced at lower temperatures (53), and hence decreased water loss in the plants. During high temperatures, wax production declines, which drastically reduces the plant's capacity to retain water and results in lethal cuticular water loss (54), which may have an adverse effect on the plants' ability to grow and develop. The significant decrease in leaf biomass observed in our study can be explained by this fatal cuticular water loss that may have a direct correlation with the decline in the growth and development of plants in high stress environments (Figure 4).

Moreover, it has been reported that the symbiotic association between *A. senegal* and its rhizobial symbiont has been inhibited above 40 °C because of the elevated root temperature. The disturbed root nodule formation may be the reason for the reduced growth in the heat-treated plants (54). Furthermore, many arid region plants like *A. senegal* undergo several morphological and anatomical adaptations that help them to survive in these extreme environmental conditions. Morphological modifications like small leaf/ leaflet structure reduce the boundary layer conductance, and anatomical modifications like a smooth or slightly rough outer cuticular surface affect leaf temperature and help the plants to survive in high temperature and less humid environments (55). We have also observed a halt in growth and shedding of leaves in H+D category from 20th day to 25th day of stress treatment. Leaf shedding and stomatal closing are a few of the various strategies adopted by plants to reduce water loss and survive in extreme hot and water deficit conditions (56).

### 3.2 ML model

Digital technologies generate a vast amount of data that necessitates higher computational and storage capabilities. Actionable insights derived from aggregated data via AI/ML techniques have tremendous real-life applications. The ML techniques are being actively employed in domains like human–computer interaction and smart healthcare. In a recent study, a multi-temporal scale aggregation refinement graph convolutional network (MTSA-RGCN) was proposed that improved the Skeleton-based human action recognition (SHAR) (57). Moreover, a structure-aware transformer-based attention fusion network (SAT-Net) was employed for the enhancement of low-quality retinal fundus images, hence improving the disease diagnostic procedure in ophthalmology (58). Similarly, many aspects of agriculture have benefited from the application of ML, particularly in the areas of leaf disease prediction (59), weed identification (60), pesticide application (61), yield prediction (62, 63), crop recognition, and crop quality. ML techniques can be used to ensure food security, safety and traceability, and crop yield monitoring, while aiding in bioactive discovery (64). In this study, we developed a custom hybrid model using CNN and SVM for the abiotic stress prediction in the leaf image dataset of 3,454 images. CNN has been used extensively in similar studies (65). To the best of our knowledge, this is the first study done for the stress prediction of bi-pinnate leaves. Several other studies have been conducted for stress or disease prediction using leaf image datasets of broad-leaf plants like banana. SIFT+ANN model proposed for the prediction of banana leaf disease achieved an accuracy score of 95% (21). Similarly, the Enhance-Nightshade-CNN model was used in model crops like tomato, potato, and eggplant to enhance the quality of nightshade crop leaf disease detection, achieving an accuracy of 95%−100% (92). In our study, the hybrid custom CNN-SVM model has proven to generate high accuracy results of 86.6% in the bipinnate leaf image dataset of *A. senegal* plant species. The model developed in our study can be extended for various other plant species to detect stress or diseases from leaf images. We have generated data on plant stress phenotyping that shows how abiotic stress is affecting the plants. Phenotyping of plant stress intensity is a crucial metric for estimating possible crop losses triggered by different abiotic and biotic stressors. It can be used to evaluate superior stress-tolerant and disease-resistant genotypes in plants, which can be employed to improve disease management strategies (66).

#### 3.2.1 Basic results

Our custom hybrid model is a fine-tuned EfficientNetB0 (CNN) and an SVM binary classifier with a Polynomial kernel for classifying *Acacia senegal* leaf images into healthy & unhealthy. The top 20 layers of EfficientNetB0 were fine-tuned, and a 1,280-dimensional feature vector was extracted, concatenated with the tabular data, and fed into an SVM classifier.

The dataset comprises 3,454 images, including 2,105 healthy samples and 1,349 stress-affected (unhealthy) samples. The per-class distribution for both the original and augmented dataset is presented in Table 1. To improve model robustness and generalization, the dataset was augmented using random horizontal/vertical flipping and rotation, increasing the effective dataset size to 16,000 images. The distribution of samples across the training, validation, and testing subsets is provided in Table 2.

**Table 1:** Per-class distribution of the original and augmented dataset.

Class	# Images in original dataset	# Images in augmented dataset
Healthy	2,105	8,000
Unhealthy	1,349	8,000

**Table 2:** Train-test-validation details.

Split	# Images in original dataset	# Images in augmented dataset	Percentage
Train	2,417	11,199	~70%
Validation	518	2,401	~15%
Test	519	2,400	~15%
Total	3,454	16,000	100%

Stratified K-fold cross-validation was employed to rigorously evaluate the model's performance and ensure its robustness across different data partitions. In this approach, the dataset is divided into *K* mutually exclusive folds, where each fold preserves the original class distribution through stratified sampling. This ensures that both healthy and unhealthy classes are represented in approximately the same proportions within each training and validation split as in the full dataset.

At each iteration, one fold is held out as the validation set while the remaining *K – 1* folds are used for training. This process is repeated *K* times, with each fold serving exactly once as the validation set. The final performance metrics are obtained by averaging the results across all folds, thereby reducing variance associated with a single train–test split and providing a more reliable estimate of generalization performance. This strategy is particularly beneficial for imbalanced datasets, as it prevents bias toward the majority class and ensures consistent evaluation across all classes.

**Figure 5:**
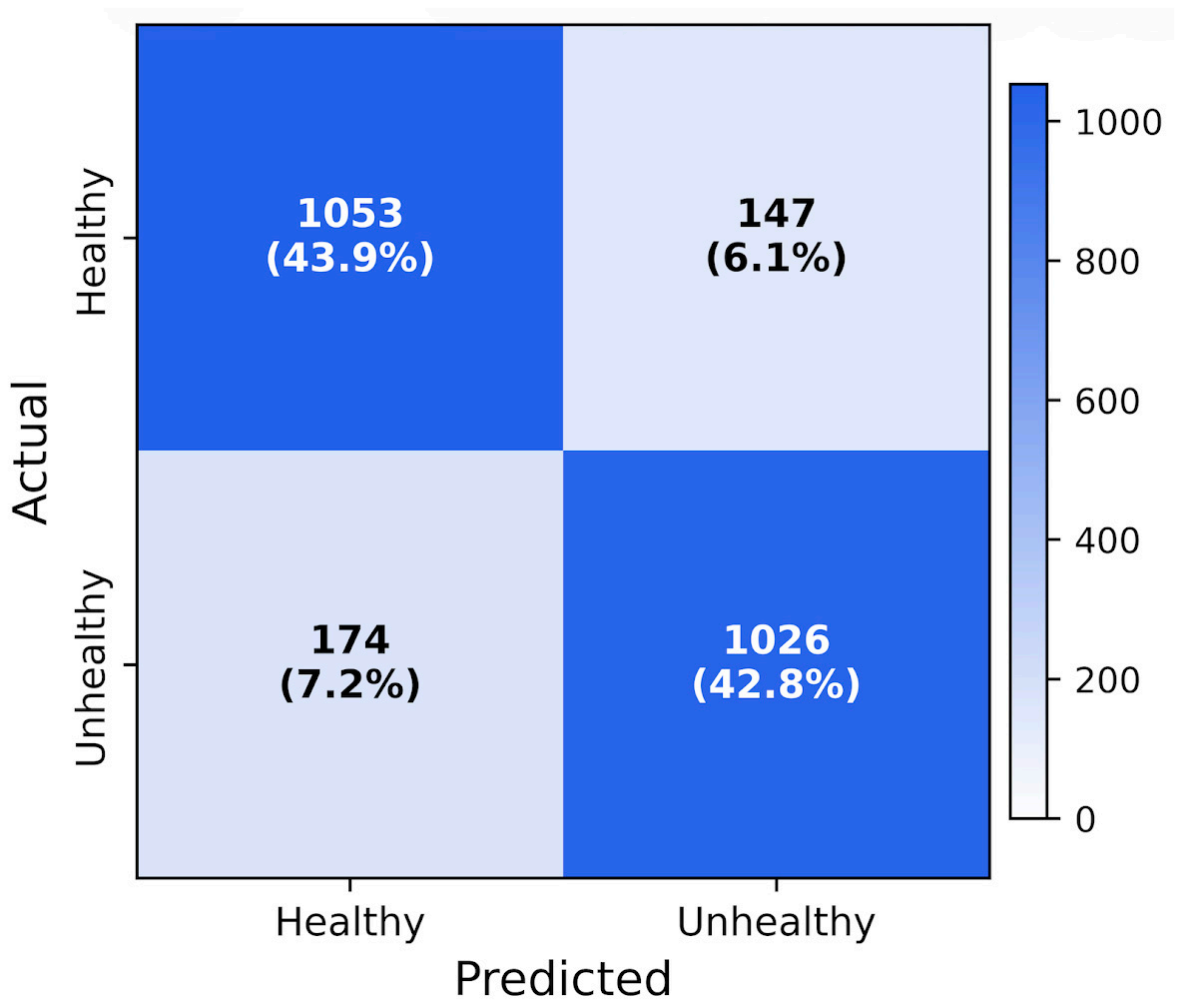
Confusion matrix of our hybrid custom CNN-SVM model.

The confusion matrix for our model is shown in Figure 5. It reflects the classification performance across the two classes, *healthy* and *unhealthy*. The diagonal entries represent correct predictions, with 1,053 Healthy samples correctly classified as Healthy and 1,026 *Unhealthy* samples correctly classified as *unhealthy*. The off-diagonal entries indicate misclassifications, comprising 147 *healthy* samples incorrectly predicted as *unhealthy* and 174 *unhealthy* samples incorrectly predicted as *healthy*. Overall, the results indicate a balanced performance of the model, with comparatively low misclassification rates and clear separability between the two classes.

**Figure 6:**
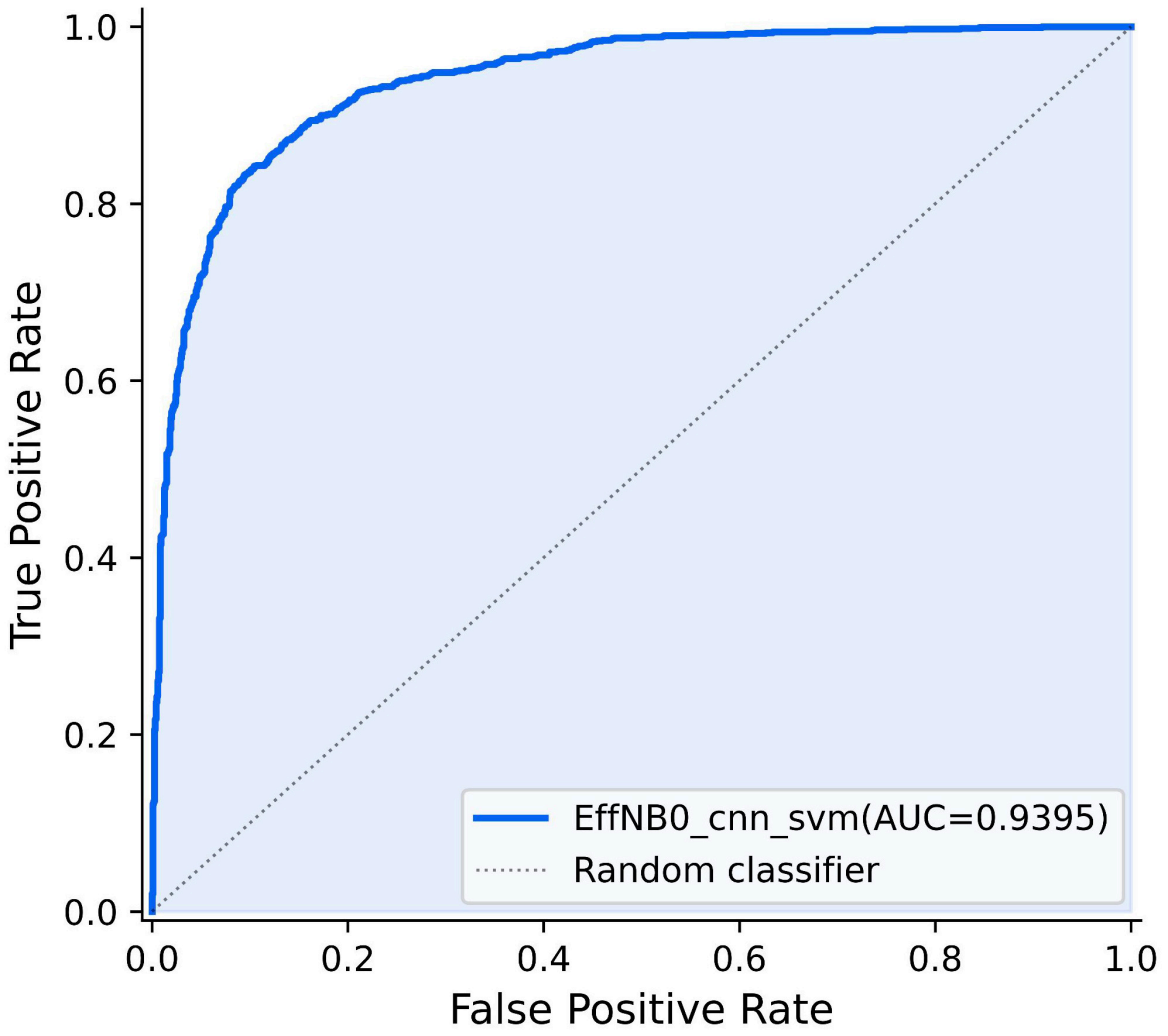
ROC curve of the proposed hybrid custom CNN_SVM model showing high discriminative performance with an AUC of 0.9395.

The ROC curve for the proposed hybrid model in Figure 6 demonstrates strong class separability across varying classification thresholds. The curve consistently lies well-above the diagonal baseline representing a random classifier, indicating that the model has a high discriminative ability in distinguishing between healthy and un*healthy Acacia senegal* leaves.

The model achieves an area under the curve (AUC) of 0.9395, reflecting strong overall performance with a high true positive rate while maintaining a low false positive rate across most threshold settings. This confirms the effectiveness of the classifier in reliably differentiating between the two classes.

In the low false positive rate region, the ROC curve shows a sharp initial rise, indicating that the model successfully identifies a large proportion of *unhealthy* samples even under strict decision thresholds. As the threshold is gradually relaxed, the true positive rate continues to increase and approaches values close to 1.0 at higher false positive rates.

Overall, the ROC analysis validates the proposed hybrid architecture as a robust and effective solution, offering strong generalization capability and a favorable balance between sensitivity and specificity across operating conditions.

**Figure 7:**
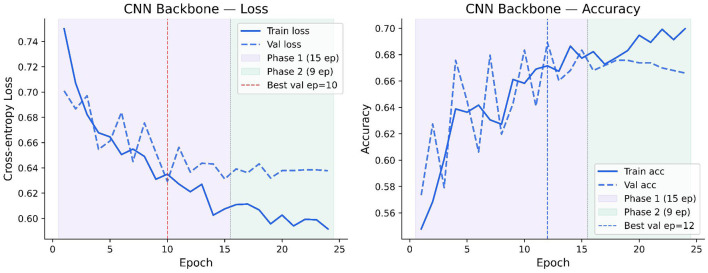
Training and validation performance of the CNN backbone over 25 epochs showing cross-entropy loss and classification accuracy across two training phases. The plots illustrate progressive convergence during Phase 1, followed by stabilized validation behavior and continued improvement in training performance during Phase 2, indicating effective learning and generally stable generalization with minor overfitting tendencies.

Figure 7 illustrates the training dynamics of the CNN backbone in terms of cross-entropy loss and classification accuracy over 25 epochs, divided into two training phases. In Phase 1, both training and validation losses show a consistent downward trend, indicating effective feature learning and stable convergence. The validation loss initially follows the training loss closely, suggesting good generalization in the early stage. The best validation performance is observed around epoch 10–12, after which improvements begin to plateau.

In Phase 2, while the training loss continues to decrease steadily, the validation loss stabilizes with minor fluctuations, indicating a slight divergence between training and validation behavior. This suggests mild overfitting, but not severe enough to degrade overall model performance. A similar trend is observed in accuracy curves, where training accuracy improves progressively up to ~0.70, while validation accuracy stabilizes around ~0.66–0.67.

Overall, the model demonstrates stable learning behavior with effective convergence and reasonable generalization. The two-phase training strategy appears beneficial in refining feature representations, while maintaining competitive validation performance across epochs.

#### 3.2.2 Ablation studies

To systematically evaluate the impact of each architecture, feature space, and data configuration, an ablation study was conducted. The baseline began with a traditional machine learning classifier (SVM) with hand-crafted features (HOG, LBP, and GLCM) on the original image dataset. Next, industry-standard pre-trained models (EfficientNetB0, DenseNet121, ResNet50) were applied to the original dataset, adapting only the final output layer for binary classification. Shifting to deep learning feature extraction provided immediate accuracy gains. Among the off-the-shelf, pre-trained architectures evaluated on the original dataset, pre-trained EfficientNetB0 outperformed both pre-trained DenseNet121 and pre-trained ResNet50 on accuracy and other key performance metrics. Consequently, it was selected for transfer learning, where it was fine-tuned on the input images of *A. senegal* leaves. This fine-tuned model became the foundational core and the custom CNN, which was used in our hybrid model, mainly to extract the final features (1280-dim), which were fed into an SVM for binary classification. This further boosted accuracy, proving that the maximum-margin boundary of the SVM was superior at separating the dense feature representations generated by the fine-tuned CNN.

The introduction of supplementary tabular features alongside the CNN embeddings yielded another notable increase in accuracy, confirming that non-visual environmental features complement raw pixel data. Finally, expanding the training environment to the augmented dataset brought the highest overall accuracy. This combinatorial data augmentation strategy effectively reduced overfitting and allowed the model to generalize across variable lighting, positioning, and noise patterns. The comprehensive summary of the empirical progression is detailed in Table 3.

**Table 3:** Performance comparison of different models/variations using evaluation metrics including AUC-ROC, Precision, Recall, F1-score, and accuracy.

Model/variation	AUC-ROC	Presicion	Recall	F1-score	Accuracy
SVM with hand-crafted features on the original dataset	0.695	0.494	0.862	0.628	0.601
Pre-trained EfficientNetB0	0.746	0.673	0.487	0.565	0.707
Pre-trained DenseNet121	0.700	0.642	0.399	0.492	0.678
Pre-trained ResNet50	0.671	0.663	0.389	0.490	0.684
Fine-tuned EfficientNetB0 (Transfer learning)	0.715	0.683	0.458	0.548	0.705
CNN + SVM	0.766	0.673	0.5271	0.591	0.714
CNN +SVM (with tabular data)	0.774	0.676	0.546	0.604	0.720
CNN +SVM (with tabular data; on augmented dataset)	0.939	0.874	0.855	0.864	0.866

For the top-performing multimodal model configuration, different SVM kernel functions were evaluated. The summary of the results is given in Table 4. The classification performance varied according to the geometric boundary definitions, with both the polynomial kernel and radial basis function (RBF) emerging as the optimal choice. The polynomial kernel was selected due to its lower computational complexity. Additionally, it maps data into a finite higher-dimensional feature space, whereas the RBF kernel operates in an infinite-dimensional space. Given identical performance metrics, the finite boundaries of the polynomial kernel offer better geometric interpretability and lower risk of overfitting on unseen test data.

**Table 4:** Performance comparison of different SVM kernels using evaluation metrics including AUC-ROC, precision, recall, F1-score, and accuracy.

Model/variation CNN +SVM (with tabular data; on augmented dataset)	AUC-ROC	Presicion	Recall	F1-score	Accuracy
RBF kernel	0.938	0.874	0.853	0.863	0.865
Linear kernel	0.802	0.736	0.685	0.710	0.720
Polynomial kernel	0.939	0.874	0.855	0.864	0.866
Sigmoid kernel	0.795	0.735	0.669	0.700	0.714

#### 3.2.3 Cross validation and confidence intervals

To rigorously evaluate the generalization capability and statistical stability of the proposed multimodal framework, a fivefold cross-validation scheme was implemented. The dataset was partitioned into five distinct folds, ensuring that each sample was utilized for both training and validation across the iterations. This process was executed across all four support vector machine (SVM) kernel variants, radial basis function (RBF), polynomial, linear, and sigmoid, to determine the most robust boundary function.

Tables 5, 6 present the individual fold area under the ROC curve (AUC) scores, along with the cross-validation mean (CV_Mean), standard deviation (CV_Std), 95% confidence intervals (CI), and independent test set metrics.

**Table 5:** Fivefold CV and test performance of CNN+SVM (with tabular data on augmented dataset) across kernels, reporting CV statistics, confidence intervals, and test metrics.

Model/Variation: CNN +SVM (with tabular data, on augmented dataset)												
Kernel	Fold1	Fold2	Fold3	Fold4	Fold5	CV_Mean	CV_Sth	CI_low	CI_high	Test_AUC	Test_Acc	Test_F1
RBF	0.921	0.925	0.919	0.930	0.919	0.923	0.005	0.918	0.927	0.938	0.865	0.864
Polynomial	0.920	0.922	0.921	0.931	0.916	0.922	0.005	0.917	0.927	0.939	0.866	0.864
Linear	0.796	0.794	0.790	0.801	0.801	0.796	0.004	0.792	0.800	0.803	0.720	0.710
Sigmoid	0.786	0.788	0.785	0.796	0.793	0.790	0.005	0.785	0.794	0.796	0.714	0.701

**Table 6:** Cross-validation mean AUC ranking and test performance of CNN+SVM (with tabular data on augmented dataset) across kernels, reported with CV mean AUC, 95% confidence interval, test AUC, and test accuracy.

CV Mean AUC ranking (all pairwise differences highly significant, *p*<0.001)				
Model/variation CNN +SVM (with tabular data) (on augmented dataset)	CV Mean AUC	95% CI	Test AUC	Test Acc
RBF kernel	0.923	0.918, 0.927	0.938	0.865
Linear kernel	0.796	0.792, 0.800	0.803	0.720
Polynomial kernel	0.922	0.917, 0.927	0.939	0.866
Sigmoid kernel	0.790	0.785, 0.794	0.796	0.714

To confirm the validity of these findings, a parametric pairwise *t*-test was performed. The analysis indicated that all pairwise differences between the models are highly statistically significant with *p* < 0.001. This confirms that the superior classification capacity of the polynomial kernel is not a byproduct of random data partitioning, but rather its innate ability to construct non-linear decision boundaries for the fused image embeddings and tabular features.

The narrow spread of the 95% confidence intervals further underscores the consistency of the proposed framework. As the polynomial kernel's confidence interval of (0.917, 0.927) shows zero overlap with the intervals of the linear (0.792, 0.800) or sigmoid (0.785, 0.794) variations, its superior classification capacity is statistically confirmed.

Additionally, the exceptionally low standard deviations across all configurations (< 0.005) demonstrate that the pre-processing pipeline, specifically the Yellow-Green (YG) filtration and data augmentation, effectively minimized variance across data splits. The independent test AUC score (0.939) for the polynomial kernel closely aligns with, and slightly exceeds, the cross-validation fold average (0.922), validating that the model generalizes well to unseen data and is protected against overfitting.

The proposed hybrid framework combines a fine-tuned EfficientNetB0 CNN with an SVM classifier to distinguish healthy and unhealthy *A. senegal* leaves using both image embeddings and tabular features. Extensive evaluation was conducted on an augmented dataset of 16,000 images using stratified fivefold cross-validation and an independent test set. The Polynomial kernel and RBF kernel SVMs achieved the best overall performance, with a CV mean AUC of ~0.922–0.923 and a test AUC of ~0.938–0.939. Linear and sigmoid kernels performed substantially worse, confirming the advantage of non-linear decision boundaries. Narrow confidence intervals and very low fold-wise variance (< 0.005) indicate strong stability and robustness across splits. The close agreement between cross-validation and test results further validates good generalization, while statistical testing (*p* < 0.001) confirms that performance differences between kernels are highly significant.

### 3.3 Biochemical analysis

#### 3.3.1 TPC assay

**Figure 8:**
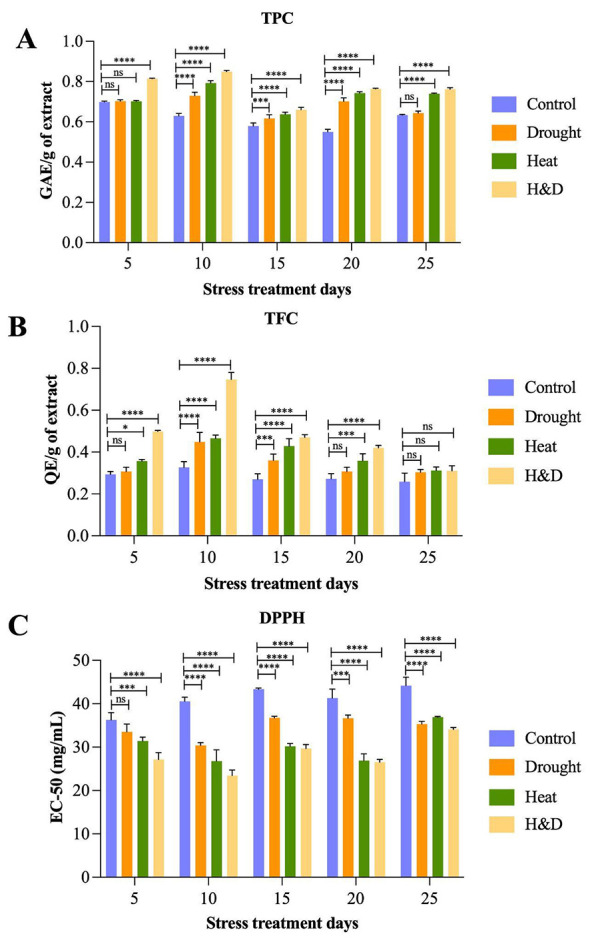
Antioxidant activities of plant leaf extracts grown in control and different stress conditions are compared at different time intervals: **(A)** Total phenolic content (TPC) calculated in gallic acid equivalent (GAE/g of extract), **(B)** Total flavonoid content (TFC) calculated in quercetin equivalent (QE/g of extract) **(C)** DPPH assay (EC_50_ values). Experiments were conducted in triplicates. Statistical significance was assessed using two-way ANOVA followed by Tukey's *post hoc* test for multiple comparisons. *p*-Value ≤ 0.05 was considered statistically significant (*p* > 0.05 ns; *p* ≤ 0.05^*^; *p* ≤ 0.001 ***; *p* ≤ 0.0001 ****). Data are presented as mean ± SD.

Production of phenolics is a response by plants against oxidative stress. The amount of phenolic compounds in plant leaf extract was measured by TPC assay. The results showed that the amount of phenolic compounds in control plants was less than the stress-treated plants at all time points (67, 68). There was a slight decrease in phenolic compounds in control plants from the 5th to 20th day, followed by an increase on the 25th day. Drought-treated plants showed less amount of phenolic compounds as compared to heat and H+D treated plants at all time point except on the 5th day. Hence, the 5th day can be considered as the initial phase of stress treatment, in which the production of phenolic compounds may have just started in heat and H+D categories, due to which all these stress-treated plants have similar phenolic content. Heat-treated plants showed slightly higher phenolic compounds than control and drought-treated plants, except on the 5th day, where an insignificant difference was observed between these categories. H+D-treated plants showed the highest amount of phenolic compounds among all the categories as well as at each time point (69). A similar study on medicinal plants like *Mentha piperita* and *Catharanthus roseus* showed an increased production of metabolites in combined stresses like H+D as compared to individual heat and drought stress (70). In all stress-treated plants, there was an increase in phenolic compounds on the 10th and 20th day compared to all other days of the stress treatment. This pattern in the graph may suggest the continuous production and degradation of the plant metabolites in response to the prolonged and extreme stress conditions. Overall, 10th day showed the best total phenolic content difference between the various stress-treated samples. The total phenolic content of control, drought, heat, and H+D-treated plants on the 10th day of the stress treatment were 0.629 ± 0.01, 0.730 ± 0.02, 0.793 ± 0.01, and 0.849 ± 0.01 mg GAE/g, respectively. H+D-treated plants showed the highest, and the control plants showed the lowest TPC values. This result is in accordance with a previously reported study, in which *Lycium shawii* (32.85 mg GAE/g) showed the highest TPC values in severe stress conditions, as compared to control plants, confirming their dependence on phenolic compounds for oxidative protection (71). Additionally, *Calligonum polygonoides* L., a stress-tolerant arid region plant, showed an increase in TPC values (64.75 ± 2.13 to 88.08 ± 0.59 mg GAE/g), with an increase in environmental temperature (72). This increase in TPC values also validates the results of our antioxidant assay (Figure 8A).

#### 3.3.2 TFC assay

TFC assay was conducted to measure the amount of flavonoids in the leaf extract. According to the results, the control plants have fewer flavonoid compounds than all the other stress-treated samples on all days (73, 74). Drought-treated plants have lesser flavonoid content than the heat and H+D-treated categories, except on the 25th day, where there is no significant difference in these stress-treated categories. This was possibly due to the degradation of plant metabolites in heat and H+D categories. Heat-treated plants showed less amount of flavonoid content as compared to the H+D-treated category, except on the 25th day. H+D-treated category showed the highest amount of flavonoids on all days (69, 75, 76). A recent study in *Solanum lycopersicum* showed a general decrease in the total flavonoid and phenolic content due to high temperature (77). All the stress-treated categories showed an increase in the amount of flavonoid compounds from the 5th day to the 10th day, followed by a decrease in all other days, except on the 25th day, where all stress-treated categories showed lesser variation in flavonoid content. This observation was possibly due to the degradation of flavonoids in the extreme conditions (77) (Figure 8B). Tenth-day samples of H+D-treated category showed the highest levels of TFC values (0.747 ± 0.03 mg QE/g), followed by heat (0.465 ± 0.02 mg QE/g), drought (0.449 ± 0.04 mg QE/g), and control (0.327 ± 0.03 mg QE/g) treated plants. The results in this study are in alignment with previous work done on a stress-tolerant arid plant species, *Calligonum polygonoides* L., which showed an increase in TFC values (2.15 ± 0.04 to 2.53 ± 0.03 mg/CtE/g) with an increase in environmental temperature (72). Additionally, a study on resilient, drought-tolerant Achillea species (*Achillea millefolium, A. nobilis, and A. filipendulina*) revealed a similar trend in TFC content, where TFC values increased with an increase in the levels of stress treatment. The TFC values of all control plants were lower than those of the stress-treated plants in all the species (68).

#### 3.3.3 LC–MS/MS analysis of plant extracts

**Figure 9:**
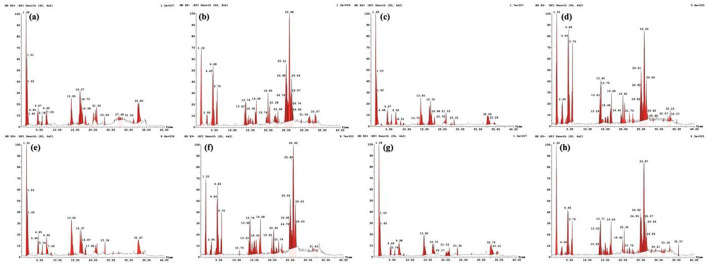
LC-MS chromatogram of methanolic extracts of control and stress-treated plant leaf extracts showing the identified peaks in positive and negative ion modes, **(a, b)** control, **(c, d)** drought-treated, **(e, f)** heat-treated, **(g, h)** H+D-treated plants.

LC-MS analysis was carried out to identify various phytochemicals such as phenolics and flavonoids from four different plant leaf samples, that is, control, drought, heat, and H+D. All the compounds identified by LC-MS analysis, both in positive and negative ionization modes, and their *m/z* values are given in Supplementary Tables S2–S5, and their respective peaks are shown in Figure 9. Phenolic and flavonoid compounds play a major role in plant development and abiotic stress tolerance (78, 79). The LC-MS analysis showed the presence of phenolic compounds like gentisic acid, gallic acid, and flavonoid compounds like (+)-Catechin gallate and Epigallocatechin gallate in all four plant leaf samples. In recent studies, the role of saturated and unsaturated fatty acids in combating heat and drought stress has been discussed (80, 81). Saturated fatty acids like pelargonic and pimelic acids were found in heat-treated plant leaf sample, whereas unsaturated fatty acids like hydroxy octadecatrienoic acid and hydroxy linolenic acid were found in the drought treated plants leaf sample. The role of phytosterols in abiotic stress tolerance in crop plants like rice has been discussed in a study, suggesting how phytosterols and their esters may play a crucial role in drought tolerance of the plant by reinforcing the cell membranes (82). Moreover, recent studies showed the increased synthesis of certain phytosterols during drought and heat stresses, indicating their significant role in abiotic stress tolerance (83). The LC-MS analysis showed the presence of phytosterols, such as Stigmasterol, a compound specific to drought-treated plant leaf samples, suggesting and validating the important role of phytosterols in the drought tolerance of the plants. Quercetin pentoside is present in the H+D sample. The phytochemical profiling of *Sorbus* L. inflorescences showed the presence of quercetin and its derivatives along with other phenolic compounds, which can be used in various industries like nutraceuticals, pharmaceuticals and cosmeceuticals (93).

#### 3.3.4 DPPH assay: antioxidant estimation assay

The phenolic/flavonoid content of plants is directly proportional to their antioxidant potential (84). According to reports, methanol (80% concentration) is the most effective solvent for extracting phenolic compounds due to its polarity and solubility of phenolic compounds (85). The DPPH assay was done to measure the antioxidant activity in the extract of plant leaves, which were subjected to different types of stress treatments. The results showed that the antioxidant activity of control plants was less than that of the stress-treated plants at all time points. Drought and heat stress led to an enhanced production of phytochemicals like phenols, flavonoids, phytosterols, and others to combat these extreme conditions (74, 86). Drought-treated plants showed less antioxidant activity as compared to heat- and H+D-treated plants in every time point except the last day, that is, the 25th day. Heat-treated plants showed less antioxidant activity than the H+D category on the 5th, 10th, and 25th days, but there was no significant difference in the heat and H+D categories on the 15th and 20th day of stress treatment. H+D category showed the highest antioxidant activity at each time point due to the increased production of metabolites like quercetin pentoside. Heat and H+D categories showed that the antioxidant activity increased on the 10th and 20th day, but comparatively decreased on the 5th, 15th, and 25th day. This trend may indicate the constant production and degradation of plant metabolites to fight against extreme environmental stress conditions. A similar trend can be seen in *Haloxylon salicornicum*, where the DPPH reaches its peak significant value at medium drought and then declines during severe drought conditions because of antioxidant system degradation (71). Day 10 showed the best antioxidant activity variation among all the stress-treated samples. The EC_50_ of control, drought, heat, and H+D treated plants on the 10th day of the stress treatment are 40.5 ± 0.9, 30.5 ± 0.8, 26.7 ± 2.5, and 23.4 ± 1.2 μg/ml. EC_50_ is the inhibition percentage, which is inversely proportional to the antioxidant activity. The EC_50_ trend on the 10th day, control < drought < heat < H+D, shows the highest antioxidant activity was achieved in H+D, while control has the least antioxidant activity. A similar trend was observed in a study, where the antioxidant activity of desert plants *Lycium shawii* (14.3%−26.4%), *Calligonum comosum* (11.3%−42.5%), and *Salvadora persica* (17.3%−28.4%) increased in response to elevated levels of abiotic stress (71).

## 4 Conclusion and future perspectives

The aim of this study is to learn more about *A. senegal*, a wild and underutilized desert plant species. Due to several agricultural, ecological, industrial, ethnomedicinal, and pharmaceutical applications of this plant, large-scale domestication can help solve the regional problems of arid and semi-arid regions like land desertification and degradation, and can also contribute to solving the global issues of food safety and security by boosting agricultural productivity.

This study presents a hybrid deep learning framework for classifying *Acacia senegal* leaf images into *healthy* and stress-affected *unhealthy* categories. The proposed model integrates a fine-tuned EfficientNetB0 convolutional neural network with a Support Vector Machine classifier, leveraging both high-dimensional image embeddings and complementary tabular features. The CNN backbone extracts a 1280-dimensional feature representation, which is used to enhance discriminative capability in the final classification stage. Extensive evaluation was performed using stratified fivefold cross-validation and an independent test set on an augmented dataset of 16,000 images. Among the evaluated kernels, the Polynomial SVM achieved the best performance, with a test AUC of 0.922 and accuracy of 86.6%, demonstrating strong generalization ability. Low variance across folds and narrow confidence intervals further confirm the model's stability and robustness. Statistical testing indicated that performance differences between kernels are highly significant (*p* < 0.001). Overall, the proposed framework provides a reliable and effective solution for plant health classification tasks. *A. senegal* is prone to a huge variety of fungal diseases, such as ceratocystis wilt disease, root rot, and heart rot (87). In the future, similar techniques can be employed for the detection of diseases.

Stress phenotyping is important to understand how various stresses impact the growth and development of plants, their morphological and physiological adaptations, which helps them to survive in extremities. Stress phenotyping shows that there was less significant difference between control and drought-treated plants compared to heat or H+D-treated plants. This showed how plants adapt to abiotic stresses through changes in allocation of biomass, morphological adaptations, and physiological regulations to sustain their growth in these extreme environmental conditions.

The biochemical analysis of these edible desert plants, subjected to different stresses, showed how stress environment leads to the production of more primary as well as secondary metabolites, and hence higher antioxidant activity and phenolic and flavonoid content. In our study, we have seen a continuous increase and decrease in the antioxidant activity, phenolic and flavonoid content among different days of the stress treatment (Figure 8). This may indicate the constant production and degradation of the metabolites in response to prolonged heat and drought stress treatments. This may indicate a mechanism by which plants in higher stress conditions combat these extreme environments by constantly generating and degrading these phytochemicals. Interestingly, researchers have seen that mild stress conditions can enhance the phytochemical profile of plants without disturbing the overall growth and yield of plants (88). This technique can be utilized to improve the variety of the plants/crops by increasing the nutritional value without affecting the growth, development, or yield of the plant. Furthermore, the phytochemical profiling of wild arid plants showed a huge variety of plant metabolites, which showed various roles in the growth, development, and protection of these plants under extreme environmental conditions. Our study paves the way for exploring more phytochemical data points that can be integrated with the developed ML framework, which can lead to easy detection of possible accumulated phytochemical compounds under abiotic stress. The phytochemicals such as phenols, flavonoids, fatty acids, and phytosterols can be used in numerous industries like cosmetics, food, beverages, and others. Future research also needs selection of better germplasm, geographical mapping, monetary investment to support such research work, and policies to promote edible wild arid plants as alternatives to mainstream crops.

## Data Availability

The plant leaf imaging data used in the work is publicly available at https://doi.org/10.5281/zenodo.16531486. The source code of the implementation is available at https://github.com/softwareinnovationslabBITS/CDRF_ASenegal_MLImaging

## Footnotes

1
https://github.com/softwareinnovationslabBITS/CDRF_ASenegal_MLImaging

## References

[1] Food and Agriculture Organization of the United Nations. The Future of Food and Agriculture: Trends and Challenges. Rome: FAO (2017).

[2] Press Information Bureau. Impact of Climate Change on Agriculture. Delhi: Press Information Bureau (2023).

[3] Savary S Bregaglio S Willocquet L Gustafson D Mason D'Croz D Sparks A . Crop health and its global impacts on the components of food security. Food Secur. (2017) 9:311–27. doi: 10.1007/s12571-017-0659-1

[4] Sõukand R. Perceived reasons for changes in the use of wild food plants in Saaremaa, Estonia. Appetite. (2016) 107:231–41. doi: 10.1016/j.appet.2016.08.011 27521164

[5] Borelli T Hunter D Padulosi S Amaya N Meldrum G Moura de Oliveira Beltrame D . Local solutions for sustainable food systems: the contribution of orphan crops and wild edible species. Agronomy. (2020) 10:231. doi: 10.3390/agronomy10020231

[6] Raddad EY Luukkanen O. The influence of different *Acacia senegal* agroforestry systems on soil water and crop yields in clay soils of the Blue Nile region, Sudan. Agric Water Manag. (2007) 87:61–72. doi: 10.1016/j.agwat.2006.06.001

[7] Musa KH Nour AH Elnour AAM Ibrahim HS Adam IM. Acacia Gums (AGs): characterization and applications. In:Elnour AAM, editor. Gum Arabic and Breast Cancer Biology: Biotechnology Perspective. Singapore: Springer (2025). pp. 1–46 doi: 10.1007/978-981-97-8518-6_1

[8] Elnour AAM Nour AH Ishag KEA. Biological applications of secondary metabolites extract (SME). from Acacia Gums (AGs). In: *Gum Arabic and Breast Cancer Biology: Biotechnology Perspective*. Singapore: Springer (2025). pp. 117–67. doi: 10.1007/978-981-97-8518-6_4

[9] Gray A Odee D Cavers S Wilson J Telford A Grant F . Does geographic origin dictate ecological strategies in *Acacia senegal* (L.). Willd? Evidence from carbon and nitrogen stable isotopes. Plant Soil. (2013) 369:479–96. doi: 10.1007/s11104-013-1593-4

[10] Omondi SF Kireger E Dangasuk OG Chikamai BN Odee DW Vandenabeele S . Genetic diversity and population structure of *Acacia senegal* (L.) Willd in Kenya. Trop Plant Biol. (2010) 3:59–70. doi: 10.1007/s12042-009-9037-2

[11] Sprent JI Odee DW Dakora FD. African legumes: a vital but under-utilized resource. J Exp Bot. (2010) 61:1257–65. doi: 10.1093/jxb/erp342 19939887

[12] Gaafar AM Salih AA Luukkanen O El Fadl MA Kaarakka V. Improving the traditional *Acacia senegal*-crop system in Sudan: the effect of tree density on water use, gum production and crop yields. Agroforestry Syst. (2006) 66:1–11. doi: 10.1007/s10457-005-2918-y

[13] Assefa A Bahiru A. Ethnoveterinary botanical survey of medicinal plants in Abergelle, Sekota and Lalibela districts of Amhara region, Northern Ethiopia. J Ethnopharmacol. (2018) 213:340–9. doi: 10.1016/j.jep.2017.11.024 29187319

[14] Barbosa FMA Cala AC Sevastyanov V Boane E Hlashwayo DF. Ethnoveterinary study of plant-based remedies for treating diseases in small ruminants in Maputo province, Mozambique. Evid Based Complement Alternat Med. (2023) 2023:1842870. doi: 10.1155/2023/1842870 37842333 PMC10569895

[15] Msimango NNP Aremu AO Amoo SO Masondo NA. Ethnoveterinary Potential of Acacia (*Vachellia and Senegalia)* Species for managing Livestock Health in Africa: from traditional uses to therapeutic applications. Plants. (2025) 14:3107. doi: 10.3390/plants14193107 41095247 PMC12526939

[16] Singh A Ganapathysubramanian B Singh AK Sarkar S. Machine learning for high-throughput stress phenotyping in plants. Trends Plant Sci. (2016) 21:110–24. doi: 10.1016/j.tplants.2015.10.015 26651918

[17] Torres-Sánchez J López-Granados F De Castro AI Peña-Barragán JM. Configuration and specifications of an unmanned aerial vehicle (UAV) for early site specific weed management. PLoS ONE. (2013) 8:e58210. doi: 10.1371/journal.pone.0058210 23483997 PMC3590160

[18] Peña JM Torres-Sánchez J Serrano-Pérez A de Castro AI López-Granados F. Quantifying efficacy and limits of unmanned aerial vehicle (UAV) technology for weed seedling detection as affected by sensor resolution. Sensors. (2015) 15:5609–26. doi: 10.3390/s150305609 25756867 PMC4435221

[19] Torres-Sánchez J Pena JM de Castro AI López-Granados F. Multi-temporal mapping of the vegetation fraction in early-season wheat fields using images from UAV. Comput Electron Agric. (2014) 103:104–13. doi: 10.1016/j.compag.2014.02.009

[20] Chandel NS Chakraborty SK Rajwade YA Dubey K. Identifying crop water stress using deep learning models. Neural Comput Appl. (2021) 33:5353–67. doi: 10.1007/s00521-020-05325-4

[21] Thiagarajan JD Kulkarni SV Jadhav SA Waghe AA Raja SP Rajagopal S . Analysis of banana plant health using machine learning techniques. Sci Rep. (2024) 14:15041. doi: 10.1038/s41598-024-63930-y 38951552 PMC11217365

[22] Harakannanavar SS Rudagi JM Puranikmath VI Siddiqua A Pramodhini R. Plant leaf disease detection using computer vision and machine learning algorithms. Glob Tran Proceed. (2022) 3:305–10. doi: 10.1016/j.gltp.2022.03.016

[23] Joshi K Awale R Ahmad S Patil S Pisal V. Plant leaf disease detection using computer vision techniques and machine learning. In: ITM web of conferences. EDP Sciences. ITM web of conferences, ICACC-2022 (2022). p. 6. doi: 10.1051/itmconf/20224403002

[24] Khalid M Sarfraz MS Iqbal U Aftab MU Niedbała G Rauf HT. Real-time plant health detection using deep convolutional neural networks. Agriculture. (2023) 13:510. doi: 10.3390/agriculture13020510

[25] Mbagwu FN Okafor VU Ekeanyanwu J. Phytochemical screening on four edible legumes (*Vigna subterranean, Glycine max, Arachis hypogea, and Vigna uniguiculata)* found in eastern Nigeria. Afr J Plant Sci. (2011) 5:370–2.

[26] Jat BL Sharma HC Pagaria P Meena AK Mali GR Khan T. Legumes: source of bioactive compounds and their potential use in legume crops improvement: a review. Legume Res. (2023) 47:1827–34. doi: 10.18805/LR-4945

[27] Martín-Cabrejas MA. Legumes: an overview (2019).

[28] Chaudhary S Dhanker R Kumar R Goyal S. Importance of legumes and role of Sulphur oxidizing bacteria for their production: a review. Legume Res. (2022) 45:275–84. doi: 10.18805/LR-4415

[29] Tomasi C Manduchi R. Bilateral filtering for gray and color images. In: Sixth international conference on computer vision (IEEE Cat. No. 98CH36271). Bombay: IEEE (1998). pp. 839–46. doi: 10.1109/ICCV.1998.710815

[30] Slinkard K Singleton VL. Total phenol analysis: automation and comparison with manual methods. Am J Enol Vitic. (1977) 28:49–55. doi: 10.5344/ajev.1977.28.1.49

[31] Tsao R Yang R Young JC Zhu H. Polyphenolic profiles in eight apple cultivars using high-performance liquid chromatography (HPLC). J Agric Food Chem. (2003) 51:6347–53. doi: 10.1021/jf0346298 14518966

[32] Wang S Meckling KA Marcone MF Kakuda Y Tsao R. Synergistic, additive, and antagonistic effects of food mixtures on total antioxidant capacities. J Agric Food Chem. (2011) 59:960–8. doi: 10.1021/jf1040977 21222468

[33] John B Sulaiman CT George S Reddy VRK. Total phenolics and flavonoids in selected medicinal plants from Kerala. Int J Pharm Pharm Sci. (2014) 6:406–8.

[34] Venuprasad MP Kandikattu HK Razack S Khanum F. Phytochemical analysis of *Ocimum gratissimum* by LC-ESI–MS/MS and its antioxidant and anxiolytic effects. S Afr J Bot. (2014) 92:151–8. doi: 10.1016/j.sajb.2014.02.010

[35] Chaudhary A Sharma S Mittal A Gupta S Dua A. Phytochemical and antioxidant profiling of Ocimum sanctum. *J Food Sci Technol*. (2020) 57:3852–63. doi: 10.1007/s13197-020-04417-2 32903995 PMC7447722

[36] Hidalgo M Sánchez-Moreno C de Pascual-Teresa S. Flavonoid–flavonoid interaction and its effect on their antioxidant activity. Food Chem. (2010) 121:691–6. doi: 10.1016/j.foodchem.2009.12.097

[37] Aroca R. Plant Responses to Drought Stress: From Morphological to Molecular Features. Berlin, Heidelberg: Springer (2012). p. 1–5 doi: 10.1007/978-3-642-32653-0

[38] Zhimin L Thompson K Spencer RE Readeer RJ. A comparative study of morphological responses of seedling roots to drying soil in 20 species from different habitats. Acta Bot Sin. (2000) 42:628–35.

[39] Zollinger N Kjelgren R Cerny-Koenig T Kopp K Koenig R. Drought responses of six ornamental herbaceous perennials. Sci Hortic. (2006) 109:267–74. doi: 10.1016/j.scienta.2006.05.006

[40] Espinoza SE Magni CR Martinez VA Ivkovic M. The effect of water availability on plastic responses and biomass allocation in early growth traits of *Pinus radiata* D. Don For Syst. (2013) 22:3–14. doi: 10.5424/fs/2013221-02084

[41] Li C. Some aspects of leaf water relations in four provenances of *Eucalyptus microtheca* seedlings. For Ecol Manage. (1998) 111:303–8. doi: 10.1016/S0378-1127(98)00336-3

[42] McMichael BL Quisenberry JE. Genetic variation for root-shoot relationships among cotton germplasm. Environ Exp Bot. (1991) 31:461–70. doi: 10.1016/0098-8472(91)90045-P

[43] Haugen R Steffes L Wolf J Brown P Matzner S Siemens DH . Evolution of drought tolerance and defense: dependence of tradeoffs on mechanism, environment and defense switching. Oikos. (2008) 117:231–44. doi: 10.1111/j.2007.0030-1299.16111.x

[44] Hausmann NJ Juenger TE Sen S Stowe KA Dawson TE Simms EL. Quantitative trait loci affecting δ13C and response to differential water availibility in Arabidopsis Thallana. *Evolution*. (2005) 59:81–96. doi: 10.1111/j.0014-3820.2005.tb00896.x 15792229

[45] Otieno DO Schmidt MWT Adiku S Tenhunen J. Physiological and morphological responses to water stress in two Acacia species from contrasting habitats. Tree Physiol. (2005) 25:361–71. doi: 10.1093/treephys/25.3.361 15631984

[46] Heredia-Guerrero JA Guzman-Puyol S Benítez JJ Athanassiou A Heredia A Domínguez E. Plant cuticle under global change: biophysical implications. Glob Chang Biol. (2018) 24:2749–51. doi: 10.1111/gcb.14276 29668107

[47] Domínguez E Heredia-Guerrero JA Heredia A. The biophysical design of plant cuticles: an overview. New Phytol. (2011) 189:938–49. doi: 10.1111/j.1469-8137.2010.03553.x 21374891

[48] Redha A Al-Mansour N Suleman P Afzal M Al-Hasan R. Leaf traits and histochemistry of trichomes of *Conocarpus lancifolius* a Combretaceae in semi-arid conditions. Am J Plant Sci. (2011) 2:165. doi: 10.4236/ajps.2011.22018

[49] Dong Z He H. Phyllode anatomy and histochemistry of four Acacia species (*Leguminosae: Mimosoideae) in* the Great Sandy Desert, north-western Australia. J Arid Environ. (2017) 139:110–20. doi: 10.1016/j.jaridenv.2017.01.001

[50] Lopez-Casado G Matas AJ DomE Cuartero J Heredia A. Biomechanics of isolated tomato (*Solanum lycopersicum* L.). fruit cuticles- the role of the cutin matrix and polysaccharides. J Exp Botany. (2007) 58:3875–83. doi: 10.1093/jxb/erm233 17975209

[51] España L Heredia-Guerrero JA Segado P Benítez JJ Heredia A Domínguez E. Biomechanical properties of the tomato (*Solanum lycopersicum*). fruit cuticle during development are modulated by changes in the relative amounts of its components. New Phytologist. (2014) 202:790–802. doi: 10.1111/nph.12727 24571168

[52] Domínguez E España L López-Casado G Cuartero J Heredia A. Biomechanics of isolated tomato (*Solanum lycopersicum)* fruit cuticles during ripening: the role of flavonoids. Func Plant Biol. (2009) 36:613–20. doi: 10.1071/FP09039 32688674

[53] Shepherd T Wynne Griffiths D. The effects of stress on plant cuticular waxes. New Phytologist. (2006) 171:469–99. doi: 10.1111/j.1469-8137.2006.01826.x 16866954

[54] Slot M Nardwattanawong T Hernández GG Bueno A Riederer M Winter K. Large differences in leaf cuticle conductance and its temperature response among 24 tropical tree species from across a rainfall gradient. New Phytol. (2021) 232:1618–31. doi: 10.1111/nph.17626 34270792 PMC9290923

[55] Fahmy GM. Leaf anatomy and its relation to the ecophysiology of some non-succulent desert plants from Egypt. J Arid Environ. (1997) 36:499–525. doi: 10.1006/jare.1996.0217

[56] Taiz L Zeiger E Møller IM Murphy A. Plant Physiology and Development. Sunderland, MA: Sinauer Associates, Inc. (2015).

[57] Li X Lu J Zhou J Liu W Zhang K. Multi-temporal scale aggregation refinement graph convolutional network for skeleton-based action recognition. Comput Animat Virtual Worlds. (2024) 35:e2221. doi: 10.1002/cav.2221

[58] Wen Y Luo B Shi W Ji J Cao W Yang X . Sat-net: structure aware transformer-based attention fusion network for low-quality retinal fundus images enhancement. In: IEEE Trans Multimedia. IEEE (2025). doi: 10.1109/TMM.2025.3565935

[59] Sarkar C Gupta D Gupta U Hazarika BB. Leaf disease detection using machine learning and deep learning: review and challenges. Appl Soft Comput. (2023) 145:110534. doi: 10.1016/j.asoc.2023.110534

[60] Xu K Shu L Xie Q Song M Zhu Y Cao W . Precision weed detection in wheat fields for agriculture 4.0: a survey of enabling technologies. methods, and research challenges. Comput Electron Agric. (2023) 212:108106. doi: 10.1016/j.compag.2023.108106

[61] Thao LQ Thien ND Bach NC Cuong DD Anh LD Khanh DG . PesViT: a deep learning approach for detecting misuse of pesticides on farm. J Supercomput. (2023) 79:15790–813. doi: 10.1007/s11227-023-05302-3

[62] Ahmed Telmoud CA Tourad MC. Harvesting the Potential of Data in Mauritania, Africa: Optimizing Machine Learning Models for Enhanced Rice Yield Prediction (2023). Available online at: https://ssrn.com/abstract=4574214 (Accessed February 2, 2026).

[63] Van Klompenburg T Kassahun A Catal C. Crop yield prediction using machine learning: a systematic literature review. Comput Electron Agric. (2020) 177:105709. doi: 10.1016/j.compag.2020.105709

[64] Joshi T Sehgal H Puri S Karnika Mahapatra T Joshi M . ML-based technologies in sustainable agro-food production and beyond: tapping the (semi) arid landscape for bioactives-based product development. J Agric Food Res. (2024) 18:101350 doi: 10.1016/j.jafr.2024.101350

[65] Abade A Ferreira PA de Barros Vidal F. Plant diseases recognition on images using convolutional neural networks: a systematic review. Comput Electron Agric. (2021) 185:106125. doi: 10.1016/j.compag.2021.106125

[66] Singh A Jones S Ganapathysubramanian B Sarkar S Mueller D Sandhu K . Challenges and opportunities in machine-augmented plant stress phenotyping. Trends Plant Sci. (2021) 26:53–69. doi: 10.1016/j.tplants.2020.07.010 32830044

[67] Jan R Kim N Lee SH Khan MA Asaf S Lubna . Enhanced flavonoid accumulation reduces combined salt and heat stress through regulation of transcriptional and hormonal mechanisms. Front Plant Sci. (2021) 12:796956. doi: 10.3389/fpls.2021.796956 34992623 PMC8724123

[68] Gharibi S Tabatabaei BES Saeidi G Goli SAH. Effect of drought stress on total phenolic, lipid peroxidation, and antioxidant activity of Achillea species. Appl Biochem Biotechnol. (2016) 178:796–809. doi: 10.1007/s12010-015-1909-3 26541161

[69] Netshimbupfe MH Berner J Van Der Kooy F Oladimeji O Gouws C. Influence of drought and heat stress on mineral content, antioxidant activity and bioactive compound accumulation in four African Amaranthus species. Plants. (2023) 12:953. doi: 10.3390/plants12040953 36840301 PMC9966708

[70] Alhaithloul HA Soliman MH Ameta KL El-Esawi MA Elkelish A. Changes in ecophysiology, osmolytes, and secondary metabolites of the medicinal plants of Mentha piperita and *Catharanthus roseus* subjected to drought and heat stress. Biomolecules. (2019) 10:43. doi: 10.3390/biom10010043 31892111 PMC7023420

[71] Amin SA Hayat F Alyafei M. Drought tolerance strategies of some native plants in the UAE desert: growth, biochemical, and antioxidant insights. BMC Plant Biol. (2025) 25:1374. doi: 10.1186/s12870-025-07305-z 41087857 PMC12522444

[72] Berwal MK Haldhar SM Ram C Shil S Kumar R Gora JS . Calligonum polygonoides L. as novel source of bioactive compounds in hot arid regions: evaluation of phytochemical composition and antioxidant activity. Plants. (2021) 10:1156. doi: 10.3390/plants10061156 34204128 PMC8229425

[73] Sarker U Oba S. Drought stress enhances nutritional and bioactive compounds, phenolic acids and antioxidant capacity of Amaranthus leafy vegetable. BMC Plant Biol. (2018) 18:258. doi: 10.1186/s12870-018-1484-1 30367616 PMC6203965

[74] Alhaithloul HAS. Impact of combined heat and drought stress on the potential growth responses of the desert grass *Artemisia sieberi* alba: relation to biochemical and molecular adaptation. Plants. (2019) 8:416. doi: 10.3390/plants8100416 31618849 PMC6843163

[75] Raja V Qadir SU Alyemeni MN Ahmad P. Impact of drought and heat stress individually and in combination on physio-biochemical parameters, antioxidant responses, and gene expression in Solanum lycopersicum. *3 Biotech*. (2020) 10:208. doi: 10.1007/s13205-020-02206-4 32351866 PMC7181466

[76] Sarker U Oba S. Drought stress effects on growth, ROS markers, compatible solutes, phenolics, flavonoids, and antioxidant activity in Amaranthus tricolor. Appl Biochem Biotechnol. (2018) 186:999–1016. doi: 10.1007/s12010-018-2784-5 29804177

[77] Alhaithloul HAS Galal FH Seufi AM. Effect of extreme temperature changes on phenolic, flavonoid contents and antioxidant activity of tomato seedlings (*Solanum lycopersicum* L). *PeerJ*. (2021) 9:e11193. doi: 10.7717/peerj.11193 34026345 PMC8123231

[78] Kumar K Debnath P Singh S Kumar N. An overview of plant phenolics and their involvement in abiotic stress tolerance. Stresses. (2023) 3:570–85. doi: 10.3390/stresses3030040

[79] Shomali A Das S Arif N Sarraf M Zahra N Yadav V . Diverse physiological roles of flavonoids in plant environmental stress responses and tolerance. Plants. (2022) 11:3158. doi: 10.3390/plants11223158 36432887 PMC9699315

[80] Ghaffari M Gholizadeh A Rauf S Shariati F. Drought-stress induced changes of fatty acid composition affecting sunflower grain yield and oil quality. Food Sci Nutr (2023) 11:7718–31. doi: 10.1002/fsn3.3690 38107128 PMC10724631

[81] He M Ding NZ. Plant unsaturated fatty acids: multiple roles in stress response. Front Plant Sci. (2020) 11:562785. doi: 10.3389/fpls.2020.562785 33013981 PMC7500430

[82] Kumar MSS Ali K Dahuja A Tyagi A. Role of phytosterols in drought stress tolerance in rice. Plant Physiol Biochem. (2015) 96:83–9. doi: 10.1016/j.plaphy.2015.07.014 26233709

[83] Mandizvo T Mabhaudhi T Mashilo J Odindo AO. Phytosterols augment endurance against interactive effects of heat and drought stress on biochemical activities of *Citrullus lanatus* var. citroides. (L. H Bailey) Mansf Ex Greb. Int J Plant Biol. (2024) 15:783–806. doi: 10.3390/ijpb15030057

[84] Joshi T Deepa PR Sharma PK. Effect of different proportions of phenolics on antioxidant potential: pointers for bioactive Synergy/Antagonism in foods and nutraceuticals. Proc Natl Acad Sci India Sec B Biol Sci. (2022) 92:939–46. doi: 10.1007/s40011-022-01396-6 35935740 PMC9340677

[85] Moteriya P Ram J Moradiya R Chanda S (2015). *In vitro* free radical scavenging and antimicrobial activity of *Cyamopsis tetragonoloba* L. J Pharmacogn Phytochem 4, 102–106.

[86] Scarano A Olivieri F Gerardi C Liso M Chiesa M Chieppa M . Selection of tomato landraces with high fruit yield and nutritional quality under elevated temperatures. J Sci Food Agric. (2020) 100:2791–9. doi: 10.1002/jsfa.10312 32022274 PMC7187367

[87] Farid AM Terhem R Aswad RM Agustini L Ho WM Indrayadi H . Diseases of Acacia and control measures in the tropics. In:Asiegbu FO Kovalchuk A, editors. Forest Microbiology. San Diego, CA: Elsevier (2023). pp. 375–400 doi: 10.1016/B978-0-443-18694-3.00012-2

[88] Oh MM Carey EE Rajashekar CB. Environmental stresses induce health-promoting phytochemicals in lettuce. Plant Physiol Biochem. (2009) 47:578–83. doi: 10.1016/j.plaphy.2009.02.008 19297184

[89] Drabo MS Van Damme EJM De Coninck T Verbeke I De Meulenaer B Savadogo A . Nutritional, phytochemical, and safety profiling of Senegalia seeds, promising wild legumes in the arid and semi-arid tropics. J Food Compos Anal. (2024) 125:105826. doi: 10.1016/j.jfca.2023.105826

[90] Ayilara MS Abberton M Oyatomi OA Odeyemi O Babalola OO. Potentials of underutilized legumes in food security. Front Soil Sci. (2022) 2:1020193. doi: 10.3389/fsoil.2022.1020193

[91] Mahapatra T Sharma P Joshi M Karnika. Acacia senegal Leaf Imaging Dataset (Version 1.0) [Dataset]. Zenodo. (2025). doi: 10.5281/zenodo.16531486

[92] Joshi BM Bhavsar H. A nightshade crop leaf disease detection using enhance-nightshade-CNN for ground truth data. Vis Comput. (2024) 40:5639–58. doi: 10.1007/s00371-023-03127-y

[93] Raudonė L Raudonis R Gaivelytė K Pukalskas A Viskelis P Venskutonis PR . Phytochemical and antioxidant profiles of leaves from different Sorbus L. species. Nat Prod Res. (2014) 28:2029–32. doi: 10.1080/14786419.2014.950577 25142632

