# Supplementary material for: Stress phenotyping of wild desert legume Acacia senegal with machine learning application and phytochemical characterization of bipinnate leaves
Source: Front Nutr. 2026 Jul 28;13:1816860. doi: 10.3389/fnut.2026.1816860 (PMC13459445; doi:10.3389/fnut.2026.1816860)
Supplement: Supplementary file 1 [file Supplementary_file_1.pdf]

Name: Nano banana
Version: Gemini 1.5 Flash
Model: Flash
Source of the generative AI technology: Google

Prompts:

Prompt 1

I have a sample architecture diagram that serves as the visual style and layout I want for my research paper. I will provide both the reference image and the complete details of my proposed methodology, including the preprocessing pipeline, model architecture, feature extraction, data flow, and training/inference workflow.

Your task is to create a new architecture diagram inspired by the overall design, clarity, and visual organization of the reference image, **not** to copy it. The diagram should accurately represent my research methodology based solely on the information I provide.

Model: CNN(EfficientNetB0)_SVM(RBF)
Input
Two parallel input streams:
Image stream — RGB leaf images
Raw shape: (16000, 224, 224, 1) float32 in [0, 1]
Converted to RGB by channel repetition: (B, 224, 224, 3) rescaled to [0, 255]
Tabular stream — 8 experimental condition columns from aug_tab.npy

Stage 1 — CNN Feature Extractor (EfficientNetB0, fine-tuned)
LayerDetailsInput(224, 224, 3)PreprocessEfficientNet preprocess_input (scales to [-1, 1])BackboneEfficientNetB0, ImageNet pretrained, include_top=FalsePoolingGlobalAveragePooling2D → 1280-dim vectorFC head (training only)Dense(256, relu)RegularizationDropout(0.3)Output (training only)Dense(1, sigmoid)

Fine-tuning procedure (2-phase, on non-augmented split_indices.npz data):
Phase 1 — 15 epochs, LR=1e-3, backbone fully frozen, only FC head trained
Phase 2 — 35 epochs, LR=1e-5, top 50 backbone layers unfrozen, rest frozen
Optimizer: Adam, loss: binary crossentropy, batch size: 16
Feature extraction (inference): The Dense/Dropout/output head is discarded. The GAP output (1280-dim) is extracted and cached to cache_features_1280.npy.

Stage 2 — Feature Fusion
ComponentDimensionalityCNN features (GAP)1280Tabular features6 Concatenated vector1286

Stage 3 — StandardScaler
StandardScaler fit on train + val indices only (idx_tv, n=13,600)
Applied to all splits (train+val for CV, test for final eval)

Stage 4 — RBF SVM Classifier
PropertyValueInput1286-dim scaled vectorKernelRBFprobabilityTrue (Platt scaling)class_weightbalancedCV strategyStratifiedKFold(n_splits=5, shuffle=True, seed=42)Param grid C[0.01, 0.1, 1, 10, 100]Param grid gamma["scale", "auto"]Selection criterionroc_aucBest paramsC=10, gamma="auto"CV AUC0.953.

The final figure should not simply be a flowchart—it should be a professional research architecture diagram that highlights the novelty and contributions of my proposed framework. It should be technically accurate, visually balanced, publication-ready, and capable of standing alongside figures in top-tier research papers.

Take the necessary time to reason through my methodology before generating the architecture. Accuracy and clarity are far more important than including every detail.

Prompt 2

Please edit the provided architecture diagram while preserving its overall layout, visual quality, and publication-ready appearance. The existing figure already has an excellent structure, so the objective is to make only the necessary modifications without altering the design philosophy or introducing unnecessary changes.

Required Modifications

1. Tabular Stream

- Modify the Tabular Stream from "8 Condition Columns" to "6 Condition Columns."
- Keep the table structure, styling, and dimensions unchanged.
- Replace the contents of the table cells with "..." placeholders instead of displaying the actual feature names, maintaining a clean and consistent appearance.

Do not modify the backbone illustration or network structure—only update the text while maintaining the same typography, spacing, and formatting.

2. Stage 4 – Classification

The classification module has changed and should be updated accordingly.

- Replace RBF SVM with Polynomial SVM.
- Remove all references and visual elements associated with the RBF kernel.
- A separate image containing the Polynomial Kernel formula is provided only as a reference. Do not merge or overlay that image into the final figure.
- Instead, recreate the mathematical expression in the same style as the existing diagram:

$$K(x_i,x_j)=\left(\gamma,x_i^\top x_j+r\right)^d$$

- The current classification block contains an RBF-style decision boundary illustration. Replace it with a clean, visually appealing illustration that better represents a Polynomial Kernel. You are free to design an appropriate nonlinear decision boundary or conceptual visualization that accurately reflects polynomial classification while matching the style of the rest of the figure.

3. Leaf Illustration

If it integrates naturally with the existing design, replace the generic leaf illustration with an Acacia senegal leaf while maintaining the same visual style and quality.

All remaining components of the architecture should remain exactly as they are. Apart from the modifications listed above, no other elements should be changed.

In particular:

- Preserve the overall layout, stage organization, color palette, icons, arrows, and spacing.
- Maintain consistent typography throughout the figure.
- Ensure all labels remain sharp, readable, and publication-quality, even when zoomed in.
- Pay special attention to Stage 1, especially the EfficientNetB0 backbone, where all layer names and annotations must remain crisp, properly aligned, and completely legible.
- Avoid overlapping text, distorted fonts, compressed labels, or any reduction in image quality.
- The final figure should retain the same professional standard as the original and be suitable for inclusion in a high-quality research publication.

The goal is to produce an updated version of the existing architecture with only the specified methodological changes while preserving its original clarity, consistency, and publication-ready quality.
