# Supplementary material for: Stress phenotyping of wild desert legume Acacia senegal with machine learning application and phytochemical characterization of bipinnate leaves
Source: Front Nutr. 2026 Jul 28;13:1816860. doi: 10.3389/fnut.2026.1816860 (PMC13459445; doi:10.3389/fnut.2026.1816860)
Supplement: Supplementary file 2 [file Table_1.docx]

**Stress phenotyping of wild desert legume *Acacia senegal* with machine learning application and phytochemical characterization of bipinnate leaves**

**Karnika Singh^1^, Rakhi Agrawal^2†^, Vighnesh PM^2^, Ankur Lhila^3^, Roshan Bagla^2^, Pankaj Kumar Sharma^1^, Mukul Joshi^1*^, Tanmaya Mahapatra^2†*^**

**Supplementary Table 1:** Review of literature for the dataset and machine learning models used for different plants, their accuracy and limitations.

| **S No.** | **Title** | **Problem Statement** | **Dataset** | **Model Used** | **Accuracy** | **Limitations** | **Reference** |
| --- | --- | --- | --- | --- | --- | --- | --- |
| 1. | **Detection of unhealthy region of plant leaves and classification of plant leaf diseases using texture features** | Identifying unhealthy regions in plant leaves and accurately classifying plant leaf diseases | Sample images of diseased plant leaves.  About 500 plant leaves of 30 different native plant species of Tamil Nadu have been collected for the approach. | Texture Analysis for feature extraction  Minimum distance classifier and SVM for classification | 94.74% | The model's generalization to diverse datasets, including unseen plants or disease types, is not discussed.  The approach heavily relies on texture-based features. Diseases that do not manifest prominently in texture changes might be challenging to detect using this method.  The paper does not address challenges like varying environmental conditions (e.g., lighting, background noise) that might affect the model's effectiveness when applied in real-world scenarios. | (Arivazhagan et al. 2013) |
| 2. | **"Analysis of Banana Plant Health Using Machine Learning Techniques"** by Joshva Devadas Thiagarajan et al. | Early Disease Detection  Automated Disease detection | **Banana LSD (Leaf Spot Disease) dataset**. The total dataset consists of **937 images** . | **ANN with SIFT (Scale-Invariant Feature Transform) Model**  **ANN with HOG (Histogram of Oriented Gradients) and LBP (Local Binary Patterns) Model** | 95% | **Invariance to Scale and Rotation**: Many existing models, especially Convolutional Neural Networks (CNNs), struggle with scale and rotation variations in images.  **Complexity and Training Data**: CNNs and other deep learning models often require large amounts of labelled training data.  **Feature Extraction Constraints**: Some feature extraction techniques, like SIFT (Scale-Invariant Feature Transform), are computationally expensive and slower compared to others like HOG (Histogram of Oriented Gradients). | (Thiagarajan et al. 2024) |
| 3. | Plant leaf disease detection using computer vision and machine learning algorithms | The problem statement emphasizes the need for a robust solution to automatically detect plant leaf diseases, specifically in tomato plants, using computer vision and machine learning techniques to assist farmers in early diagnosis and prevention of crop losses. | **Plant Village Dataset**, which contains images of tomato leaves affected by six different disorders. | Feature Extraction using DWT, PCA, GLCM  Classifier used is CNN | 99.09% | Variations in leaf colour due to environmental effects (e.g., lighting, weather conditions) can make disease identification more difficult.  The model is trained and tested on the **Plant Village Dataset**, which may not represent all real-world scenarios or varieties of plant diseases.  The segmentation process (e.g., K-means clustering) may struggle with complex leaf images or overlapping features. | (Harakannanavar et al. 2022) |
| 4. | **"Real-Time Plant Health Detection Using Deep Convolutional Neural Networks"** by Khalid et al., published in *Agriculture* in 2023. | The problem statement of the paper focuses on the challenge of detecting plant diseases in real-time to minimize crop losses and improve agricultural productivity. | Exclusive dataset: This dataset contains images of **money plant leaves** that were collected in a controlled environment.  Public Dataset: The public dataset used in the study is the **Plant Village Dataset** from Kaggle, which includes images of **bell pepper** and **potato** leaves. | YOLOv5 | 93% | Despite the high accuracy, there were still instances where the model failed to correctly detect certain diseased leaves or produced false positives/negatives.  The accuracy of the model is influenced by the quality of the datasets used. In this research, the datasets were captured using mobile cameras, which may not offer the same resolution or quality as high-end imaging equipment  The exclusive dataset, in particular, had a relatively small sample size, which could limit the model's ability to generalize well to unseen data. | (Khalid et al. 2023) |
| 5. | **"A Review on Plant Leaf Diseases Detection and Classification Based on Machine Learning Models" by Sripada Swain, Sasmita Kumari Nayak, and Swati Sucharita Barik.** | The paper focuses on reviewing various machine learning models and techniques, including image preprocessing, segmentation, feature extraction, and classification methods, to detect and classify plant leaf diseases effectively. | None | None | None | **Limited Dataset Diversity**  **Dependence on Image Quality**  **Computational Complexity** | (Swain et al. 2020) |
| 6. | **"Detection of Plant Diseases and Nutritional Deficiencies from Unhealthy Plant Leaves Using Machine Learning Techniques" by Debolina Nath, Dr. Pushan Kumar Dutta, and Anup Kumar Bhattacharya.** | The paper aims to address this issue by leveraging image processing and machine learning techniques to automatically detect plant diseases and predict nutritional deficiencies from images of unhealthy plant leaves. | The paper does not explicitly mention using a specific publicly available dataset for its research. Instead, it discusses the methodology of processing images of diseased plant leaves, focusing particularly on rice crops. | Segmentation using K means clustering  SVM for classification | Not mentioned | **Limited Dataset and Generalization**  **Dependence on Image Quality** | (Nath et al. 2021) |
| 7. | **"Detection of Unhealthy Citrus Leaves Using Machine Learning Techniques"** | **Early Detection of Citrus Leaf Diseases**  **Manual Limitations**  **Need for Automated Solutions** | The dataset of healthy and unhealthy 100 leaves is acquired from mendeley repository. | SVM/MLP | MLP - 74.8%  SVM - 61.6% | The dataset size is too less  Accuracy is low | (Goyal et al. 2022) |
| 8. | **Proximal Methods for Plant Stress Detection Using**  **Optical Sensors and Machine Learning** | The problem statement of the paper centers around the need for innovative and efficient methods to detect plant stresses, which significantly impact crop yields and global food security. | The dataset used in this paper includes various sources of plant health data, specifically focusing on agricultural stress detection. The authors discuss the use of **RGB images** from smartphones and digital cameras, as well as **hyperspectral images** and **fluorescence imaging**, for identifying and classifying plant stress | SVMs and ANNs | None(Review paper) | ANNs and deep learning models, especially convolutional neural networks (CNNs), require significant computational resources.   The SVM model, while effective in high-dimensional data, can be impacted by noise, making it challenging to define a clear decision boundary. | (Zubler and Yoon 2020) |
| 9. | **Machine Learning for Plant Leaf Disease**  **Detection and Classification – A Review** | The problem statement in this paper highlights the difficulty and importance of detecting plant leaf diseases, which significantly affect crop yield and economic value | None  (Review Paper) | SVM  ANN  CNN | None | **Dataset Challenges**: Lack of large, diverse, and high-quality datasets for training models.  **Environmental Factors**: Variations in lighting, background, and environmental conditions during image capture can reduce the accuracy of models. | (Annabel et al. 2019) |
| 10. | **Estimating Yield for Fruit Trees Using Image Processing and Artificial Neural Network** | The paper addresses the challenge of estimating fruit yield for trees before harvesting. Traditional methods of assessing fruit yield are labour-intensive, time-consuming, and prone to human error, especially when managing large-scale orchards | The dataset used in the study consists of **150 images of orange trees**, captured in the **Serik district of Antalya, Turkey**, which is well-known for its orange production. | ANN model based on MLP | 89.8% | **The dataset is relatively small, with only 150 images from 20 trees**  **Images were taken using ordinary cameras, such as cellphone cameras, which may not capture the fine details of the fruit or tree structure needed for more accurate predictions.** | (Yaşar and Akdemir 2017) |
| 11. | **A New Deep Learning System for Wild Plants Classification and Species Identification: Using Leaves and Fruits** | The study aims to develop a system using artificial intelligence, particularly deep learning, to automate and enhance the process of plant identification. | Images of wild plants were collected from their natural habitat in Egypt.  The dataset consists of **51 species** from two plant families:  Apiaceae (8 species)  Brassicaceae (9 species). | CNN  SVM  RF | Categorical classification:  98.2%(CNN)  96.7%(SVM)  96%(RF)  Binary Classification:  99.7%(RF)  99.5%(SVM)  99%(CNN) | The dataset size is relatively small.  Lower accuracy for certain plant parts, such as upper leaves, compared to fruits and lower leaves. | (Ibrahim et al. 2021) |
| 12. | **Multiple Regression Models and Computer Vision Systems to Predict Antioxidant Activity and Total Phenols in Pigmented Carrots** | The paper addresses the challenge of developing a non-destructive, efficient, and cost-effective approach to predict the nutritional quality of pigmented carrots (e.g., antioxidant activity and total phenols) using a **Computer Vision System (CVS)** | The study uses four types of carrots (orange, light orange, purple, and yellow) collected from Polignano a Mare, Southern Italy. The dataset includes:  60 carrots for model development.  24 carrots for model validation. | Linear Regression | **R² = 0.97** for antioxidant activity (AA).  **R² = 0.94** for total phenols (TP)  **R² = 0.93** for AA.  **R² = 0.86** for TP | **Uneven Pigmentation: Lower accuracy in predicting AA and TP for internal parts of carrots with uneven pigmentation compared to external parts.**  **Colorimeter vs. CVS: The colorimeter-based predictions were less reliable, especially for unevenly pigmented samples.** | (Pace et al. 2013) |
| 13 | **Comparative Analysis of Machine**  **Learning and Evolutionary**  **Optimization Algorithms for**  **Precision Micropropagation of**  **Cannabis sativa: Prediction and**  **Validation of in vitro Shoot Growth**  **and Development Based on the**  **Optimization of Light and**  **Carbohydrate Sources** | To predict shoot length, root length, nodes, shoots are canopy area from light intensity and sucrose concentrations in cannabis sativa L. | 264 plantlets were used to collect data on shoot length and canopy surface area. | MLP, Generalized regression neural network, adaptive neuro-fuzzy inference system | Coefficient of Determination of GRNN was 0.964 for shoot length, 0.714 for shoot number, 0.744 for nodes, 0.914 for root length and 0.944 for canopy surface | **Experiment conducted in closed environment which may be impacting results and showing possible correlations. If the same experiment is conducted with forced air or open lids, high dependence of plants on sucrose may reduce.** | (Pepe et al. 2021) |
| 14 | **Machine Learning Techniques for Predicting Crop**  **Photosynthetic Capacity from Leaf Reflectance**  **Spectra** | To Evaluate the potential  of leaf reflectance spectroscopy to predict parameters of photosynthetic capacity in Brassica oleracea  and Zea mays | 40 plants per genus |  |  |  | (Heckmann et al. 2017) |
| 15 | **Spatiotemporal Responses of Vegetation to Hydroclimatic**  **Factors over Arid and Semi-arid Climate** | Understanding the dynamics of vegetative greenness and how it interacts with various  hydroclimatic factors is crucial for comprehending the implications of global climate change | Normalized difference vegetation index for over 21 years (2001-2021) in Rajasthan India. Rainfall, Land Surface Temperature and Evapo Transpiration were also used | NA | Study provides evidence of degraded lands in Rajasthan | **Only Mann Kendall’s trend test was used to evaluate changes. Other methodologies could have been used to take an average and provide a holistic statistical analysis** | (Yadav et al. 2023) |
| 16 | **Rooting in the Desert: A Developmental Overview on**  **Desert Plants** | Review paper  Discussion of how desert plants have adapted their root system architecture to cope with water and nutrient scarcity in deserts | NA | NA | NA | **-**NA | (Kirschner et al. 2021) |
| 17 | **Kumar, S. and Singh, N., 2009. Micropropagation of Prosopis cineraria (l.) Druce–a multipurpose desert tree. *Researcher*, *1*(3), pp.28-32.** | Developing an efficient protocol for micropropagation of *Prosopis cineraria*, a drought-resistant and economically valuable desert tree | Nodal explants (approximately 1-1.3 cm long) were obtained from a mature tree growing in ,Kurukshetra university | NA | NA | **NA** | (Kumar and Singh 2009) |
| 18 | **Finely-grained annotated datasets for image-based plant phenotyping** | Lack of finegrain phenotyping datasets to compare existing methodologies | [Dataset generation study] | NA | NA | **NA** | (Minervini et al. 2016) |
| 19 | **Machine learning prediction of mortality in the common desert shrub**  **Encelia farinosa** | Assessing and predicting mortality patterns of *Encelia farinosa* shrubs at different life-history stages (seedling, yearling, and mature adult) in arid ecosystems of the Mojave Desert | Annual surveys of two populations of farinosa shrubs in mojave desert since 1981. Their height, width along widest access and width along perpendicular axis was one set of features along with extent of canopy leave cover and flowering | Random Forest, logistic regression and plain statistics | 89% in Death Valley using random forest and 84% using logistic regression while the accuracies fall to 74 and 70 percent respectively in Oatman | **Other algorithms could have been explored to provide a better view** | (Bitter and Ehleringer 2021) |
| 20 | **Phenolic Profiles and Antioxidant Activity of**  **Germinated Legumes** | This study aims to investigate the changes in phenolic content, identify individual phenolic compounds, and assess antioxidant activity in six legume species during a five-day germination period. | The study analysed six legume species:  **Black Beans (Vigna cylindrica)**  **Mung Beans (Vigna radiata)**  **Peanuts (Arachis hypogaea)**  **Adzuki Beans (Vigna angularis)**  **Soybeans (Glycine max)**  **White Cowpeas (Vigna unguiculata)** | Linear Regression Analysis | **DPPH Scavenging Activity**: Strongest correlation with sinapic acid (R² = 0.965).  **Reducing Power**: Strongest correlation with ellagic acid (R² = 0.910). | **Species-Specific Observations: The findings are specific to the six legumes studied and may not generalize to other crops.**  **Focus on Germination: The study only examined phenolic changes during germination, excluding other growth stages.** | (Khang et al. 2016) |
| 21 | **Comparison of phenolic profiles, antioxidant capacity and relevant enzyme**  **activity of different Chinese wheat varieties during germination** | The study addresses this issue by analysing changes in phenolic profiles, antioxidant capacity, and enzyme activity in three Chinese wheat varieties during germination to explore their relationships and potential applications. | The study used **three Chinese wheat varieties**:  **Jimai 22**  **Sumai 188**  **Luyuan 502**  The grains were harvested in 2015 from Shandong and Jiangsu provinces in China | PCA  MLRA | **R² = 0.965** for bound phenolic content.  **R² = 0.964** for DPPH antioxidant capacity.  **R² = 0.951** for ABTS antioxidant capacity. | **Generalizability: The study focuses only on three specific Chinese wheat varieties, limiting the application of findings to other wheat types or crops.**  **Complex Phenolic Biosynthesis: The diversity and temporal expression of phenolic compounds make it challenging to generalize relationships across different conditions or species.** | (Chen et al. 2017) |
| 22 | **Antioxidant and anti-inflammatory properties of Prosopis cineraria based**  **phenolic rich ointment in wound healing** | Study the potential wound healing activity of  BFPC | Healthy adult male wistar albino rats weighing 180–200 g were  utilized | NA | NA | **Study shows correlation of wound healing and BFPC but the exact cause is indeterminate** | (Yadav et al. 2018) |
| 23 | **Machine Learning Approaches to Improve Three Basic**  **Plant Phenotyping Tasks Using Three-Dimensional**  **Point Clouds** | Challenges in automated processing of large 3D point cloud datasets for plant phenotyping, specifically lamina/stem classification, lamina counting, and stem skeletonization | 54 full shoot architechtures were used. 36 tomato plants and 18 Nicotiana benthamiana plants.  Point feature and fast point feature histograms used to get features | SVM, KNN, Random Forest | 95% accuracy in stem vs lamina classification  86.60% accuracy in lamina counting  95% precision in stem skeletonization | **Only applicable to isolate leaves of tomato plants as it is based on structure of tomato plants** | (Ziamtsov and Navlakha 2019) |

**Supplementary Table 2:** LC–MS/MS data of control plant sample.

| **S. No.** | **Predicted compounds** | **Mol. wt.** | **m/z** | **Retention time (RT)** | **Peak ID** | **Citations** |
| --- | --- | --- | --- | --- | --- | --- |
| 1 | L-Phenylalanine | 165.2 | 103.0, 120.0, 166.9 | 25.08 | 77 | (Abdallah et al. 2021) |
| 2 | 2-Amino-1,3,4-octadecanetriol | 317.9 | 256.2, 300.2, 318.1 | 15.54 | 42 | (Abdallah et al. 2021) |
| 3 | (+)-Catechin gallate | 442.2 | 123.0, 151.0 | 25.15 | 78 | (Abdallah et al. 2021) |
| 4 | Gallic acid | 171 | 125.0, 169.0 | 1.51 | 3 | (Abdallah et al. 2021) |
| 5 | Epigallocatechin gallate | 458.3 | 125.0, 169.0, 165.2 | 18.7 | 53 | (Abdallah et al. 2021) |
| 6 | Gentisic acid | 154.9 | 109.0, | 24.74 | 73 | (Abdallah et al. 2021) |
| 7 | Quercetin pentoside/**Quercetin-3-D-xyloside** | 433.1 | 255.1, 254.1, | 5.78 | 22 | (Perestrelo et al. 2012) |
| 8 | Naringin | 579.2 | 564.2, 520.1, 476.4, 433.1, 417, 378.4, 291.3, 271.4 | 5.59 | 20 | (Zhang et al. 2011a) |
| 9 | Lariciresinol 4-*O*-glucoside | 359.4 | 347.2, 329.2, 307.3, 190.9, 180.0, 175.1, 160.9 | 26.04 | 82 | (Hanhineva et al. 2012) |
| 10 | Diosmetin | 607.3 | 217.0, 180.0,332.1, 317.9, 304.0, 261.2, | 32.62 | 111 | (Zhang et al. 2011b) |
| 11 | Esculin | 339.2 | 180.0, 179.9, 177.0, | 15.54 | 42 | (Tattini et al. 2014) |
| 12 | Docosenoic acid/Erucic acid | 339.3 | 338.2, 321.1, 297.3, 296.3, 282.3, 261.2, 250.0, 240.1, 222.9, 203.0, | 15.76 | 44 | (Dunn et al. 2012) |

**Supplementary Table 3:** LC–MS/MS data of Drought treated plant sample.

| **S. No.** | **Predicted compounds** | **Mol. Wt.** | **m/z** | **Retention time (RT)** | **Peak ID** | **Citations** |
| --- | --- | --- | --- | --- | --- | --- |
| 1 | L-Phenylalanine | 165.1 | 103.0,107.0,120.0,166.1 | 25.08 | 77 | (Abdallah et al. 2021) |
| 2 | 2-Amino-1,3,4-octadecanetriol | 317.2 | 318.3, 256.2, 300.3 | 15.54 | 42 | (Abdallah et al. 2021) |
| 3 | (+)-Catechin gallate | 442.2 | 123.0,139.0,151.0,153.0 | 25.15 | 78 | (Abdallah et al. 2021) |
| 4 | gallic acid | 171 | 125.0, 169.0 | 1.51 | 3 | (Abdallah et al. 2021) |
| 5 | Epigallocatechin gallate | 457.1 | 125.0, 169.0, 165.1 | 18.7 | 53 | (Abdallah et al. 2021) |
| 6 | Gentisic acid | 154.8 | 109.0, 153.0, | 24.74 | 73 | (Abdallah et al. 2021) |
| 7 | Quercetin pentosyl hexoside/Reynoutrin | 595.2 | 535.4, 463.5, 433.1, 377.1, 353.4, 329.5, 301.1 | 2.84 | 10 | (Liu et al. 2012) |
| 8 | Quercetin pentoside/**Quercetin-3-D-xyloside** | 433.1 | 385.2, 301.1, 299.0, 297.3, | 5.78 | 22 | (Perestrelo et al. 2012) |
| 9 | Naringin | 579.2 | 564.2, 520.3, 476.4, 433.1, 417.2, 291.5, 271.1 | 5.59 | 20 | (Zhang et al. 2011a) |
| 10 | Docosenoic acid/ Erucic acid | 339.3 | 338.4, 321.1, 297.3, 282.3, 261.2, 250.1, 240.2, 222.0, 203.1 | 15.54 | 50 | (Dunn et al. 2012) |
| 11 | 7-hydroxycoumarin | 160.9 | 157.0, | 14.28 | 45 | (Han-Qing et al. 2019) |
| 12 | Kaempferitrin | 563.3 | 531.3, 471.2, 435.3, 431.1, 417.2, 297.3, 285.2, 269.3 | 4.29 | 15 | (Kolniak-Ostek et al. 2013) |
| 13 | Oxo-octadecadienoic acid/13-oxo-9Z,11E-octadecadienoic acid | 293.1 | 281.3, 269.3, 236.1, 220.4, 207.4, 201, 190.9, 174.9 | 5.27 | 22 | (Dunn et al. 2012) |
| 14 | Esculin | 339.2 | 179.9, 177.1 | 28.49 | 103 | (Tattini et al. 2014) |
| 15 | Hydroxy linolenic acid | 293.2 | 257.3, 235.2, 225.2, 210.1, 190.9, 174.9 | 30.99 | 111 | (Dunn et al. 2012) |
| 16 | Hydroxy octadecatrienoic acid/9(*S*)-HOTrE | 293.2 | 271.1, 243.2, 213.0, 174.9, 157.0, | 25.01 | 84 | (Dunn et al. 2012) |
| 17 | Stigmasterol/NSC 8095 | 413.2 | 391.4, 359.5, 338.3, 301.1, 279.1, 261, 222.0, 188.9, 167.0 | 6.92 | 28 | (Rozenberg et al. 2003) |

**Supplementary Table 4:** LC–MS/MS data of heat-treated plant sample.

| **S.No.** | **Predicted compounds** | **Mol. Wt.** | **m/z** | **Retention time (RT)** | **Peak ID** | **Citations** |
| --- | --- | --- | --- | --- | --- | --- |
| 1 | L-Phenylalanine | 165.1 | 120.9 | 25.08 | 77 | (Abdallah et al. 2021) |
| 2 | 2-Amino-1,3,4-octadecanetriol | 317 | 256.2, | 15.54 | 42 | (Abdallah et al. 2021) |
| 3 | (+)-Catechin gallate | 442.2 | 123.0,139.0,151.4,153.0 | 25.15 | 78 | (Abdallah et al. 2021) |
| 4 | Gallic acid | 171 | 125.0, 169.0 | 1.51 | 3 | (Abdallah et al. 2021) |
| 5 | Epigallocatechin gallate | 458.4 | 125.0, 169.0, 165.0 | 18.7 | 53 | (Abdallah et al. 2021) |
| 6 | Gentisic acid | 154.8 | 109.0, 153.0, | 24.74 | 73 | (Abdallah et al. 2021) |
| 7 | Docosenoic acid/ Erucic acid | 339.2 | 338.9, 321.3, 297.3, 296.4, 282.3, 261.3, 250.1, 240.2, 222.1, 203.1 | 15.54 | 50 | (Dunn et al. 2012) |
| 8 | Myristoleic acid | 227.2 | 217.0, 213.1, 209.1, 197.1, 182.9 | 19.9 | 66 | (Dunn et al. 2012) |
| 9 | Nonanoic acid/Pelargonic acid | 158.9 | 154.9, 151.4, 149.6 | 19.4 | 64 | (ZHU and MATSUMOTO 1987) |
| 10 | Pimelic acid | 158.9 | 155.1, 150.8 | 32.57 | 116 | (Taamalli et al. 2015) |

**Supplementary Table 5:** LC–MS/MS data of heat+drought treated plant sample.

| **S.No.** | **Predicted compounds** | **Mol wt.** | **m/z** | **Retention time (RT)** | **Peak ID** | **Citations** |
| --- | --- | --- | --- | --- | --- | --- |
| 1 | L-Phenylalanine | 165 | 107.0,120.9,166.9 | 25.08 | 77 | (Abdallah et al. 2021) |
| 2 | 2-Amino-1,3,4-octadecanetriol | 318.2 | 318.9, 256.2, | 15.54 | 42 | (Abdallah et al. 2021) |
| 3 | (+)-Catechin gallate | 442.1 | 123.0,139.9,151.0,153.0 | 25.15 | 78 | (Abdallah et al. 2021) |
| 4 | gallic acid | 171.1 | 125.0, 169.0 | 1.51 | 3 | (Abdallah et al. 2021) |
| 5 | Epigallocatechin gallate | 458.1 | 125.0, 169.0, 165.1, 305.4 | 18.7 | 53 | (Abdallah et al. 2021) |
| 6 | Gentisic acid | 154.8 | 109.0, 153.0, | 24.74 | 73 | (Abdallah et al. 2021) |
| 7 | Quercetin pentoside/ **Quercetin-3-D-xyloside** | 433.2 | 403.1, 385.3, 301.2, 299.4, 297.2, | 5.78 | 22 | (Perestrelo et al. 2012) |
| 8 | Naringin | 579.2 | 564.1, 520.3, 476.4, 433.2, 417.4, 291.3, 271.1 | 5.59 | 20 | (Zhang et al. 2011a) |
| 9 | Umbelliferone/7-hydroxycoumarine | 160.9 | 157.0, | 14.28 | 45 | (Han-Qing et al. 2019) |
| 10 | Myristoleic acid | 227.2 | 217.0, 213.3, 209.1, 197.1, 182.9 | 19.54 | 65 | (Dunn et al. 2012) |
| 11 | Docosenoic acid/Erucic acid | 339.3 | 338.2, 321.1, 297.2, 296.2, 282.3, 261.2, 250.0, 222.0, 203.1 | 15.54 | 50 | (Dunn et al. 2012) |
| 12 | Esculin | 339.0, | 180.1, 179.1, 177.6 | 5.56 | 23 | (Tattini et al. 2014) |

**References**

Abdallah MS, Mustafa M, Nallappan MA, et al (2021) Determination of phenolics and flavonoids of some useful medicinal plants and bioassay-guided fractionation substances of Sclerocarya birrea (A. Rich) Hochst stem (bark) extract and their efficacy against Salmonella typhi. Front Chem 9:670530

Annabel LSP, Annapoorani T, Deepalakshmi P (2019) Machine learning for plant leaf disease detection and classification–a review. In: 2019 international conference on communication and signal processing (ICCSP). IEEE, pp 538–542

Arivazhagan S, Shebiah RN, Ananthi S, Varthini SV (2013) Detection of unhealthy region of plant leaves and classification of plant leaf diseases using texture features. Agricultural Engineering International: CIGR Journal 15:211–217

Bitter NQ, Ehleringer JR (2021) Machine learning prediction of mortality in the common desert shrub Encelia farinosa. Ecol Inform 64:101376

Chen Z, Wang P, Weng Y, et al (2017) Comparison of phenolic profiles, antioxidant capacity and relevant enzyme activity of different Chinese wheat varieties during germination. Food Biosci 20:159–167

Dunn SR, Thomas MC, Nette GW, Dove SG (2012) A lipidomic approach to understanding free fatty acid lipogenesis derived from dissolved inorganic carbon within cnidarian-dinoflagellate symbiosis. PLoS One 7:e46801

Goyal N, Kumar S, Saraswat M (2022) Detection of unhealthy citrus leaves using machine learning technique. In: 2022 12th International Conference on Cloud Computing, Data Science & Engineering (Confluence). IEEE, pp 591–595

Hanhineva K, Rogachev I, Aura A-M, et al (2012) Identification of novel lignans in the whole grain rye bran by non-targeted LC–MS metabolite profiling. Metabolomics 8:399–409

Han-Qing W, Sha-Sha W, Qi W (2019) Umbelliferone ameliorates renal function in diabetic nephropathy rats through regulating inflammation and TLR/NF-κB pathway. Chin J Nat Med 17:346–354

Harakannanavar SS, Rudagi JM, Puranikmath VI, et al (2022) Plant leaf disease detection using computer vision and machine learning algorithms. Global Transitions Proceedings 3:305–310

Heckmann D, Schlüter U, Weber APM (2017) Machine learning techniques for predicting crop photosynthetic capacity from leaf reflectance spectra. Mol Plant 10:878–890

Ibrahim NMA, Gabr DG, Emara A-HM (2021) A new deep learning system for wild plants classification and species identification: Using leaves and fruits. In: International Conference of Reliable Information and Communication Technology. Springer, pp 26–37

Khalid M, Sarfraz MS, Iqbal U, et al (2023) Real-time plant health detection using deep convolutional neural networks. Agriculture 13:510

Khang DT, Dung TN, Elzaawely AA, Xuan TD (2016) Phenolic profiles and antioxidant activity of germinated legumes. Foods 5:27

Kirschner GK, Xiao TT, Blilou I (2021) Rooting in the desert: A developmental overview on desert plants. Genes (Basel) 12:709

Kolniak-Ostek J, Oszmiański J, Wojdyło A (2013) Effect of l-ascorbic acid addition on quality, polyphenolic compounds and antioxidant capacity of cloudy apple juices. European food research and technology 236:777–798

Kumar S, Singh N (2009) Micropropagation of Prosopis cineraria (l.) Druce–a multipurpose desert tree. Researcher 1:28–32

Liu T, Liu Z, Song C, et al (2012) Chitin-induced dimerization activates a plant immune receptor. Science (1979) 336:1160–1164

Minervini M, Fischbach A, Scharr H, Tsaftaris SA (2016) Finely-grained annotated datasets for image-based plant phenotyping. Pattern Recognit Lett 81:80–89

Nath D, Dutta PK, Bhattacharya AK (2021) Detection of plant diseases and nutritional deficiencies from unhealthy plant leaves using machine learning techniques. In: 4th Smart Cities Symposium (SCS 2021). IET, pp 351–356

Pace B, Cefola M, Renna F, et al (2013) Multiple regression models and computer vision systems to predict antioxidant activity and total phenols in pigmented carrots. J Food Eng 117:74–81

Pepe M, Hesami M, Small F, Jones AMP (2021) Comparative analysis of machine learning and evolutionary optimization algorithms for precision micropropagation of Cannabis sativa: Prediction and validation of in vitro shoot growth and development based on the optimization of light and carbohydrate sources. Front Plant Sci 12:757869

Perestrelo R, Lu Y, Santos SAO, et al (2012) Phenolic profile of Sercial and Tinta Negra Vitis vinifera L. grape skins by HPLC–DAD–ESI-MSn: Novel phenolic compounds in Vitis vinifera L. grape. Food Chem 135:94–104

Rozenberg R, Ruibal-Mendieta NL, Petitjean G, et al (2003) Phytosterol analysis and characterization in spelt (Triticum aestivum ssp. spelta L.) and wheat (T. aestivum L.) lipids by LC/APCI-MS. J Cereal Sci 38:189–197

Swain S, Nayak SK, Barik SS (2020) A review on plant leaf diseases detection and classification based on machine learning models. Mukt shabd 9:5195–5205

Taamalli A, Arráez‐Román D, Abaza L, et al (2015) LC‐MS‐based metabolite profiling of methanolic extracts from the medicinal and aromatic species Mentha pulegium and Origanum majorana. Phytochemical analysis 26:320–330

Tattini M, Di Ferdinando M, Brunetti C, et al (2014) Esculetin and esculin (esculetin 6-O-glucoside) occur as inclusions and are differentially distributed in the vacuole of palisade cells in Fraxinus ornus leaves: A fluorescence microscopy analysis. J Photochem Photobiol B 140:28–35

Thiagarajan JD, Kulkarni SV, Jadhav SA, et al (2024) Analysis of banana plant health using machine learning techniques. Sci Rep 14:15041

Yadav B, Malav LC, Singh S V, et al (2023) Spatiotemporal responses of vegetation to hydroclimatic factors over arid and semi-arid climate. Sustainability 15:15191

Yadav E, Singh D, Yadav P, Verma A (2018) Antioxidant and anti-inflammatory properties of Prosopis cineraria based phenolic rich ointment in wound healing. Biomedicine & Pharmacotherapy 108:1572–1583

Yaşar GH, Akdemir B (2017) Estimating yield for fruit trees using image processing and artificial neural network. International Journal of Advances in Agricultural & Environmental Engg(IJAAEE) 4:8–11

Zhang M, Duan C, Zang Y, et al (2011a) The flavonoid composition of flavedo and juice from the pummelo cultivar (Citrus grandis (L.) Osbeck) and the grapefruit cultivar (Citrus paradisi) from China. Food Chem 129:1530–1536

Zhang M, Duan C, Zang Y, et al (2011b) The flavonoid composition of flavedo and juice from the pummelo cultivar (Citrus grandis (L.) Osbeck) and the grapefruit cultivar (Citrus paradisi) from China. Food Chem 129:1530–1536

ZHU XR, MATSUMOTO K (1987) Absorption and Translocation of 6-Benzylamino Purine in Satsuma [Citrus unshiu Marc.] Trees. Journal of the Japanese Society for Horticultural Science 56:159–165

Ziamtsov I, Navlakha S (2019) Machine learning approaches to improve three basic plant phenotyping tasks using three-dimensional point clouds. Plant Physiol 181:1425–1440

Zubler A V, Yoon J-Y (2020) Proximal methods for plant stress detection using optical sensors and machine learning. Biosensors (Basel) 10:193
